# CD95-Mediated Calcium Signaling Promotes T Helper 17 Trafficking to Inflamed Organs in Lupus-Prone Mice

**DOI:** 10.1016/j.immuni.2016.06.028

**Published:** 2016-07-19

**Authors:** Amanda Poissonnier, Doriane Sanséau, Matthieu Le Gallo, Marine Malleter, Nicolas Levoin, Roselyne Viel, Lucie Morere, Aubin Penna, Patrick Blanco, Alain Dupuy, Florence Poizeau, Alain Fautrel, Julien Seneschal, Florence Jouan, Jerome Ritz, Edouard Forcade, Nathalie Rioux, Cécile Contin-Bordes, Thomas Ducret, Anne-Marie Vacher, Paul A. Barrow, Robin J. Flynn, Pierre Vacher, Patrick Legembre

**Affiliations:** 1 Centre Eugène Marquis, Rue Bataille Flandres Dunkerque, 35042 Rennes, France; 2 INSERM ERL440-OSS, Equipe Labellisée, Ligue Contre Le Cancer, 35042 Rennes, France; 3 Université de Rennes 1, 2 Ave. du Prof. Léon Bernard, 35043 Rennes, France; 4 Bioprojet Biotech, Rue du Chesnay Beauregard, 35760 Saint-Grégoire, France; 5 Biosit, Plateforme H2P2, Biogenouest, 2 Ave. du Prof. Léon Bernard, 35043 Rennes, France; 6 INSERM U1085, 2 Ave. du Prof. Léon Bernard, 35043 Rennes, France; 7 Université de Bordeaux, CHU Bordeaux, 146 Rue Léo Saignat, 33076 Bordeaux, France; 8 CNRS UMR 5164, 146 Rue Léo Saignat, 33076 Bordeaux, France; 9 Centre Hospitalier Universitaire Rennes, 2 Rue Henri Le Guilloux, 35022 Rennes, France; 10 INSERM U1035, 146 rue Léo Saignat, 33076 Bordeaux, France; 11 Division of Hematologic Malignancies and Department of Medical Oncology, Dana-Farber Cancer Institute, Boston, MA 02115, United States; 12 INSERM U1045, 146 rue Léo Saignat, 33076 Bordeaux, France; 13 INSERM U1218, Institut Bergonié, 33076 Bordeaux, France; 14 School of Veterinary Medicine and Science, University of Nottingham, Leicestershire LE12 5RD, United Kingdom

**Keywords:** Calcium, CD95, inflammation, lupus, sphingosine-1 phosphate, Th17 cell

## Abstract

CD95 ligand (CD95L) is expressed by immune cells and triggers apoptotic death. Metalloprotease-cleaved CD95L (cl-CD95L) is released into the bloodstream but does not trigger apoptotic signaling. Hence, the pathophysiological role of cl-CD95L remains unclear. We observed that skin-derived endothelial cells from systemic lupus erythematosus (SLE) patients expressed CD95L and that after cleavage, cl-CD95L promoted T helper 17 (Th17) lymphocyte transmigration across the endothelial barrier at the expense of T regulatory cells. T cell migration relied on a direct interaction between the CD95 domain called calcium-inducing domain (CID) and the Src homology 3 domain of phospholipase Cγ1. Th17 cells stimulated with cl-CD95L produced sphingosine-1-phosphate (S1P), which promoted endothelial transmigration by activating the S1P receptor 3. We generated a cell-penetrating CID peptide that prevented Th17 cell transmigration and alleviated clinical symptoms in lupus mice. Therefore, neutralizing the CD95 non-apoptotic signaling pathway could be an attractive therapeutic approach for SLE treatment.

## Highlights

• CD95-mediated Ca^2+^ response promotes endothelial transmigration of Th17 cells
• CD95 interacts with PLCγ1 to induce Ca^2+^ response and Th17 cell migration
• Ca^2+^ response stems from a CD95 region different from death domain
• Inhibition of the CD95-mediated Ca^2+^ response alleviates disease in lupus-prone mice

## Graphical Abstract

**Figure:**
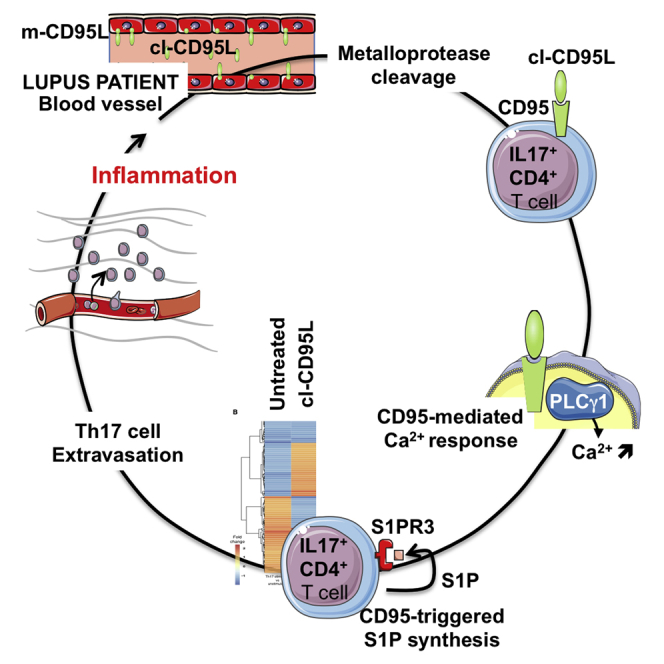

## Teaser

Cleaved CD95L (cl-CD95L) does not trigger apoptosis, and its pathophysiological role remains unclear. Legembre and colleagues demonstrate that CD95L is expressed in SLE patient blood vessels and that after cleavage, cl-CD95L recruits Th17 cells to inflamed organs. In lupus-prone mice, injection of a molecule blocking cl-CD95L function alleviates signs of disease.

## Introduction

CD95 ligand (CD95L, also known as FasL) is a transmembrane glycoprotein that acts locally through cell-to-cell contact (Suda et al., 1993). The extracellular domain of CD95L comprises a juxtamembrane stalk region (Orlinick et al., 1997) that is cleavable by metalloproteases (Fouqué et al., 2014), and this cleavage releases CD95L into the bloodstream. CD95 (also known as Fas, APO-1, and TNFRSF6) belongs to the tumor necrosis factor receptor (TNF-R) family and is ubiquitously expressed in the body (Peter et al., 2015). When membrane-bound CD95L binds to CD95, the intracellular region of CD95 (designated the death domain [DD]) orchestrates the formation of a death-inducing signaling complex (DISC) by recruitment of the adaptor molecule, Fas-associated protein with death domain (FADD), which in turn induces caspase-8 aggregation and subsequent apoptosis (Kischkel et al., 1995). By contrast, we and others have shown that metalloprotease-cleaved CD95L (cl-CD95L) promotes formation of an atypical molecular complex designated motility-inducing signaling complex (MISC) that stimulates non-apoptotic signaling pathways and increases intracellular calcium (Ca^2+^) content (Kleber et al., 2008, Malleter et al., 2013, Tauzin et al., 2011).

Systemic lupus erythematosus (SLE) is a chronic autoimmune disorder of largely unknown etiology; its pathogenesis can affect almost all organs and tissues. Human studies and murine models indicate a role for IL-17-producing T helper 17 (Th17) cells in SLE progression (see (Shin et al., 2011) for a review). Lupus-prone mice are partially protected from immunopathology by a reduction in renal Th17 cell accumulation (Steinmetz et al., 2009). Therefore, abnormal Th17 cell trafficking to inflamed organs might promote SLE pathogenesis, and modulation of Th17 cell migration is an attractive therapeutic option for reducing disease-related inflammation. However, the precise mechanism of Th17 cell accumulation in damaged SLE organs remains unclear.

A close relationship exists between SLE pathogenesis and deregulation of CD95 signaling already. Patients suffering from autoimmune lymphoproliferative syndrome (ALPS) type Ia harbor CD95 mutations responsible for lymphoproliferation and SLE-like autoimmunity (Drappa et al., 1996, Fisher et al., 1995, Rieux-Laucat et al., 1995). These ALPS type Ia patients show heterozygous mutations (Straus et al., 2001) that lead to the inhibition of the CD95-mediated apoptotic signal without affecting activation of non-apoptotic signaling pathways (Legembre et al., 2004). Accordingly, we hypothesize that aggravation of lupus symptoms is caused not only by a defective apoptotic process (Kühtreiber et al., 2003) but also by induction of CD95-mediated non-apoptotic signaling pathways.

Herein, we demonstrate that high concentrations of cl-CD95L in SLE patients promoted Th17 cell migration across the endothelial barrier at the expense of regulatory T (Treg) cells in a Ca^2+^-dependent manner. This CD95-mediated Ca^2+^ signaling occurred independently of the CD95-DD; exploiting this result, we designed a therapeutic molecule that selectively neutralized the CD95-mediated non-apoptotic signaling pathways. Moreover, in a murine model of SLE, this treatment halted the pro-inflammatory actions of cl-CD95L, alleviating both immune and pathological hallmarks of SLE.

## Results

### CD95L in SLE Serum Promotes Endothelial Transmigration of Activated Th17 Cells

**Figure 1:**
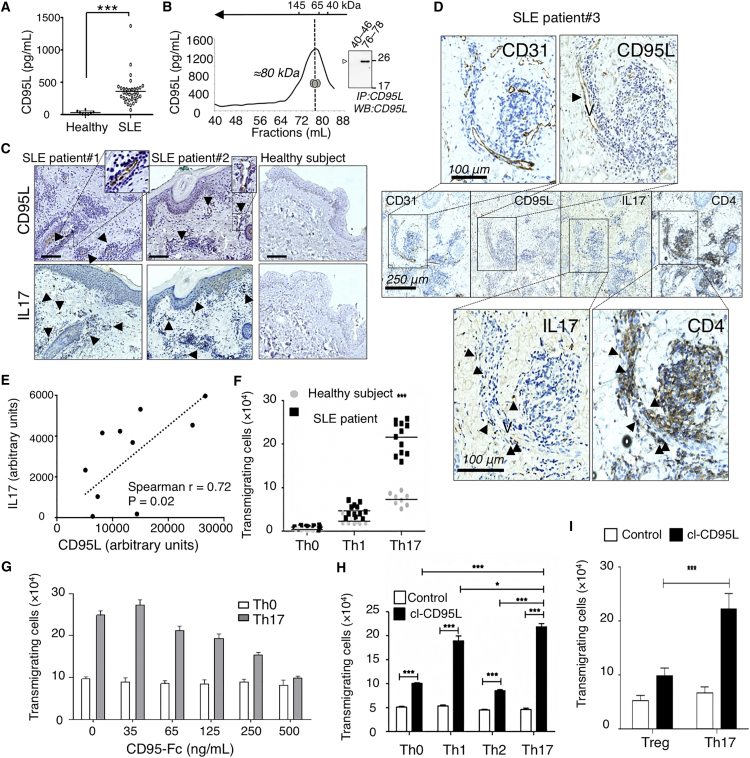
Serum CD95L in SLE Patients Induces Transmigration of T Lymphocytes (A) Soluble CD95L levels were measured in serum from newly diagnosed SLE patients (n = 34) and healthy donors (n = 8) via ELISA (Student’s t test). (B) CD95L in SLE serum was fractionated by size-exclusion column chromatography and measured via ELISA. *Inset*: CD95L was immunoprecipitated from gel-filtration fractions 40–46 and 76–78 and subjected to immunoblotting. The image is representative of gel-filtration analysis of four different patients. (C) CD95L and IL-17 staining in inflamed skin samples from lupus patients or healthy mastectomy subjects. Numbers represent different patients. The scale bar represents 100 μm. (D) CD95L, CD31, CD4, and IL-17 staining in inflamed skin samples from an SLE patient. “V” represents an endothelial vessel, and arrowheads identify marker-expressing cells. (E) Densitometric analysis of CD95L and IL-17 staining in different patients. (F) Transmigration of human T cell subpopulations in the presence of serum from SLE patients or healthy donors. (G) Th17 T cell transmigration in the presence of SLE serum containing the indicated concentrations of CD95-Fc. Undifferentiated Th_0_ T cells served as controls. Data represent means ± SD of five individual serum donors. (H) CD4^+^ T cell transmigration with or without cl-CD95L (100 ng/mL). Data represent means ± SD of three independent experiments. (I) Treg and Th17 cell transmigration with or without cl-CD95L (100 ng/mL) Data represent means ± SD of three independent experiments (two-way ANOVA).

Soluble CD95L concentrations in serum obtained from SLE patients were higher than those from age-matched healthy donors (Figure 1A). SLE serum was fractionated by size-exclusion chromatography (Figure 1B). By ELISA, CD95L was mainly detected in fractions 76–78; the proteins in these fractions showed molecular masses ranging from 75 to 80 kDa under native conditions. Next, CD95L was immunoprecipitated from these fractions, and denaturing conditions revealed a polypeptide band of 26 kDa (Figure 1B). These results do not rule out the possibility that soluble CD95L is associated with another partner but do strongly suggest that serum CD95L in SLE patients corresponds to a homotrimeric ligand.

We next hypothesized that if serum CD95L contributes to inflammatory processes in SLE patients by promoting extravasation of Th17 cells in inflamed tissues, then CD95L-expressing cells should be detected in these tissues and surrounded by IL-17-expressing cells. Therefore, we performed an immunohistochemical analysis of skin biopsies obtained from SLE patients to examine the distribution of CD95L- and IL-17-expressing cells. Skin biopsies from SLE patients, but not from healthy controls, showed positive staining for CD95L and IL-17 (Figure 1C and Figure S1 in the Supplemental Information available online). CD95L expression was mainly restricted to blood-vessel endothelial cells, and these cells were surrounded by infiltrating immune cells (Figure 1C and Figure S1). Serial slices of skin biopsies from SLE patients revealed that IL17 was co-expressed by CD4^+^ T cells (Figure 1D), indicating that CD95L-expressing blood vessels were surrounded by infiltrating Th17 cells. Moreover, a densitometric analysis of stained tissues from lupus patients (n = 10) revealed a correlation between CD95L concentrations and the number of tissue-infiltrating, IL-17-expressing immune cells (Figure 1E). This suggested that CD95L might behave as a chemoattractant for Th17 cells.

To examine whether after cleavage by metalloproteases, soluble CD95L had a chemoattractive effect on all T cells or was selective for distinct Th cell subsets, we next evaluated transmigration of undifferentiated (Th0) and differentiated (Th1 and Th17) CD4^+^ T cells with or without serum from SLE patients. Compared with healthy serum, SLE serum triggered a moderate increase in Th1 cell transmigration and a marked increase in Th17 cell transmigration (Figure 1F). Pre-incubation of SLE serum with a decoy CD95 receptor (CD95-Fc) dose dependently inhibited Th17 cell migration, indicating that transmigration of these cells relied on CD95 signaling (Figure 1G).

We produced a homotrimeric, metalloprotease-cleaved version of human CD95L (cl-CD95L) (Tauzin et al., 2011). Similar to CD95L in SLE serum, cl-CD95L more efficiently promoted the transmigration of Th1 and Th17 lymphocytes relative to undifferentiated Th0 and differentiated Th2 cells (Figure 1H). Because an imbalance between the Th17/Treg cell ratio in inflamed organs has been previously reported in the pathogenesis of SLE and other autoimmune disorders (Yang et al., 2009), we also examined the effect of cl-CD95L on Treg cell transmigration. Cl-CD95L increased the endothelial transmigration of in-vitro-differentiated Th17 cells, but not that of Treg cells (Figure 1I). These results support the idea vthat high concentrations of serum CD95L in SLE patients can cause pro-inflammatory Th17 cell accumulation and destabilize Th17/Treg cell balance in diseased organs.

To determine the mechanism responsible for this preferential transmigration of Th17 cells, we examined the expression of adhesion molecules known to promote endothelial transmigration. Cl-CD95L had no effect on the expression concentrations of adhesion molecules expressed by human umbilical-vein endothelial cells (HUVECs) (Figure S1B). On the other hand, although stimulation of Th17 cells with cl-CD95L had no effect on the amount of lymphocyte function-associated antigen-1 (LFA-1, the ICAM-1 binding partner), it led to upregulation of P-selectin glycoprotein-1 (PSGL-1, an E-/P-selectin ligand) (Figure S1C). The concentration of PSGL-1 was also upregulated in Th1 cells but to a lesser extent than in Th17 cells, and it tended to be downregulated in Treg cells (Figure S1C). Because HUVECs used in our transmigration assay expressed E-selectin but not P-selectin (Figure S1B), we next examined whether blocking these E-selectin-PSGL-1 interactions could abrogate the CD95-mediated Th17 transmigration. An E-selectin-neutralizing monoclonal antibody (mAb) inhibited Th17 cell transmigration but did not affect the weak endothelial transmigration observed in Treg cells exposed to cl-CD95L (Figure S1D). This antibody was more effective in preventing the CD95-mediated endothelial transmigration of Th17 cells than of Th1 cells (Figure S1D). To understand why Th1 cells responded less efficiently to cl-CD95L than Th17 cells, we compared the quantity of CD95 in different Th subsets from 20 blood donors. Although the amount of CD95 was not lower in Treg cells than in Th17 cells (Figure S1E), Th1 cells exhibited a lower concentration of CD95 than did Th17 cells (Figure S1E). This finding might then explain why, in the presence of cl-CD95L, Th1 cells migrated with a lower intensity than Th17 cells.

**Figure 2:**
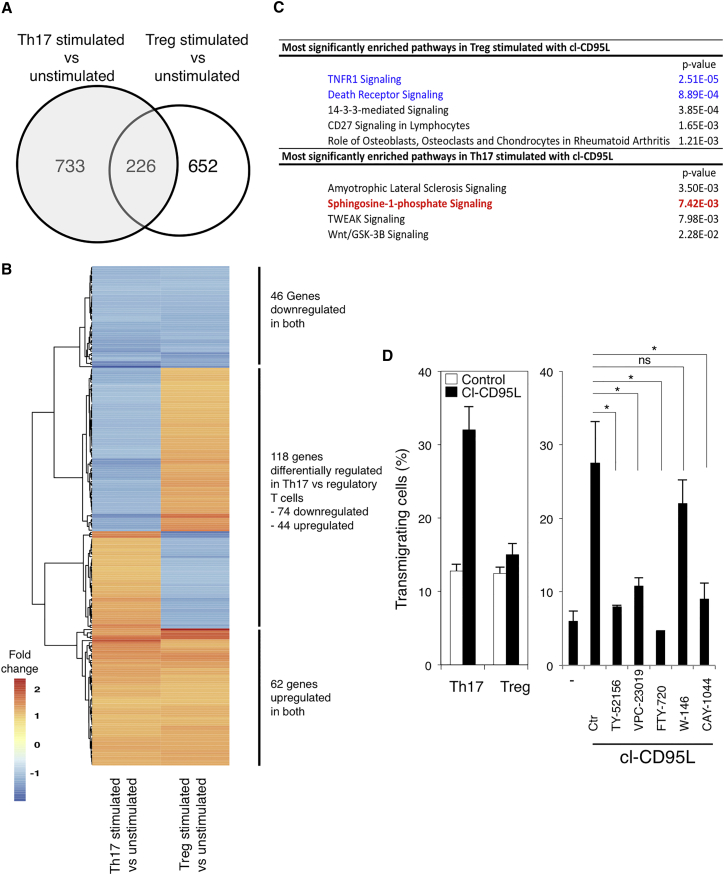
Transcriptomic Signature in Human Th17 and Treg Cells Stimulated with cl-CD95L (A) Venn diagram comparing the genes differentially expressed between untreated and cl-CD95L-treated T cell subsets. (B) Heat map depicting the relative n-fold change in the amount of transcripts significantly (p ≤ 0.05) and differentially expressed between Th17 and Treg cells stimulated with cl-CD95L (100 ng/mL). Data for each experimental group (n = 2 per condition) are shown. The color gradient indicates n-fold change, as shown. (C) Pathway enrichment analysis of genes whose expression is significantly modulated by cl-CD95L in Th17 and Treg cells and associated p values. (D) *Left panel*: endothelial transmigration of human Th17 and Treg cells was evaluated in the presence or absence of cl-CD95L (100 ng/mL) in the Boyden chamber. *Right panel*: Th17 cells were pre-treated with FTY720 (1 μM), TY-52156 (10 μM), VPC-23019 (10 μM), W146 (1 μM), or CAY-1044 (1 μM) and then exposed to cl-CD95L (100 ng/mL), and endothelial transmigration was evaluated via the Boyden chamber. Data represent means ± SD of three independent experiments.

To address how cl-CD95L selectively promoted the endothelial transmigration of Th17 cells to the detriment of Treg cells, we investigated how cl-CD95L stimulation affected the gene expression profile of Th17 and Treg cells. Human Th17 cells (CD4^+^CXCR3^−^CCR6^+^CD45RA^−^) and Treg cells (CD4^+^CD127^low^CD25^+^) were sorted from the blood of two different donors (Figure S1F) and stimulated in the presence or absence of cl-CD95L. Transcriptomic signatures of Treg and Th17 cells stimulated with cl-CD95L were analyzed (Figures 2A and 2B and Tables S1 and S2). Gene ontology analysis revealed that the sphingosine 1 phosphate (S1P) signaling pathway was modulated in Th17 cells stimulated with cl-CD95L (Figure 2C), whereas Treg cells exposed to cl-CD95L showed overexpression of genes responsible for the implementation of cell death (Figure 2C). The S1P signaling pathway (Figure S1G) can promote endothelial transmigration of activated T and B lymphocytes, leading to extra- or intravasation. Using Th17 and Treg cells sorted as aforementioned, we confirmed that cl-CD95L promoted endothelial transmigration of Th17 cells but not Treg cells (Figure 2D). Pre-incubation of Th17 cells with FTY720, a chemical that induces internalization of four S1P receptors (S1P-1, -3, -4, and -5) (Brinkmann et al., 2002) indicated that cl-CD95L selectively promoted endothelial transmigration of Th17 cells by stimulating the S1P signaling pathway (Figure 2D). To evaluate the role of S1PR3 in the CD95-mediated Th17 transmigration, we next tested the effect of additional antagonist molecules, namely TY-52156 (Murakami et al., 2010), CAY-1044, VPC-23019, and W-146 (Li et al., 2015) (Figure 2D), on Th17 cell migration. Although the selective S1PR3 inhibitors, TY-52156 and CAY-1044, and the S1PR1 and S1PR3 inhibitor VPC-23019 efficiently inhibited the passage of Th17 cells across endothelial cells (Figure 2D), the S1PR1 antagonist W146 did not prevent CD95-mediated Th17 transmigration (Figure 2D). Overall, these findings established that cl-CD95L promotes a selective Th17 cell transmigration that partially relies on the increased PSGL-1-E-selectin interactions, the production of S1P, and the implementation of the S1PR3-driven signaling pathway.

### Cl-CD95L Recruits Th17 Cells In Vivo

**Figure 3:**
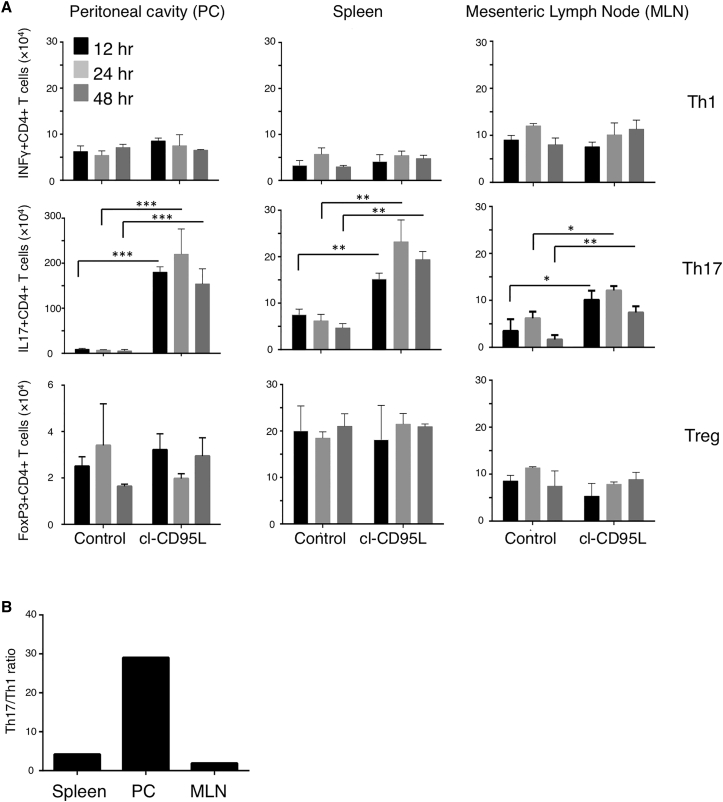
Cl-CD95L Is a Chemoattractant for Th17 Cells In Vivo Mice received a single injection of cl-CD95L (200 ng/animal) or vehicle and were sequentially sampled. (A) T cell populations were obtained from tissues or peritoneal cavity washes. Cells were then restimulated in the presence of PMA and ionomycin for 4 hr and then analyzed by flow cytometry. Cells were identified as follows; Th1 (CD4^+^IFN-γ^+^), Th17 (CD4^+^IL-17^+^), and Treg (CD4^+^Foxp3^+^). Numbers of infiltrating cells were calculated. (B) Ratios of Th17/Th1 cells per organ were determined 24 hr after injection. Data represent two independent experiments with six mice/group; means ± SEM are displayed.

To demonstrate in vivo that cl-CD95L is a chemoattractant for Th17 cells, we injected C57BL/6 mice intraperitoneally with cl-CD95L and examined infiltrating cells in the peritoneal cavity, spleen, and mesenteric lymph nodes (MLNs) 12, 24, and 48 hr later. We evaluated the number of activated CD4^+^ T cells recruited to these organs in cl-CD95L-injected mice by using loss of CD62L as an indication of T cell activation. While the total number of Th1 and Treg cells did not change in the peritoneal cavity, spleen, or MLNs of cl-CD95L-injected mice as compared to mice administered control medium (Figure 3A), Th17 cells accumulated in the peritoneal cavity and, to a lesser extent, in peripheral secondary lymphatic organs, such as spleen and MLN, of cl-CD95L-injected mice (Figures 3A and 3B). Real-time quantitative polymerase chain reaction (qPCR) analysis of key Th17 lineage markers (IL-17, IL-23R, and C-C chemokine receptor type 6 [CCR6]) of activated CD4^+^ T cells confirmed that cl-CD95L recruited Th17 cells to these tissues (Figures S2A and S2C). No increase was observed in the number of cells expressing IFN-γ (indicative of Th1 cells) or FoxP3 (indicative of Treg cells) (Figures S2D and S2E), confirming that injection of cl-CD95L in the peritoneal cavity of immune-competent mice creates a cl-CD95L gradient that promotes the endothelial transmigration of Th17 cells.

### CD95 Triggers a DD-Independent Ca^2+^ Response

**Figure 4:**
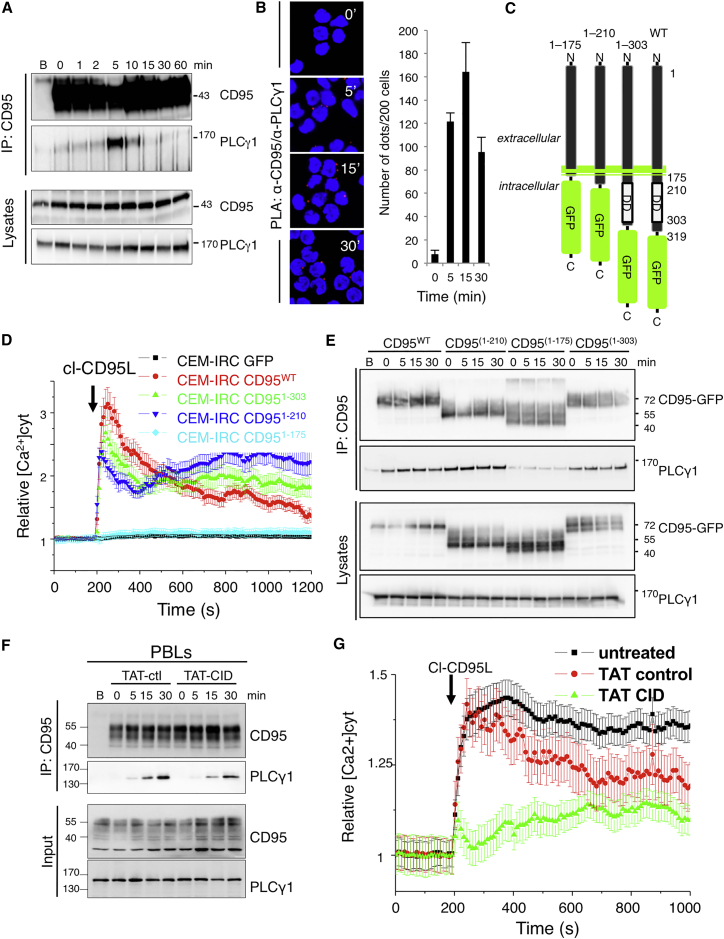
CD95 Induces a DD-Independent Ca^2+^ Response (A) CEM T cells were stimulated with cl-CD95L (100 ng/mL). Cells were lysed, and CD95 was immunoprecipitated. The protein complex was resolved by SDS-PAGE and subjected to immunoblotting. Total lysates served as controls. The column marked “B” indicates treatment with beads alone. Data are representative of three independent experiments. (B) *Left panel*: Th17 cells from peripheral blood were stimulated with cl-CD95L (100 ng/mL) for indicated times. PLA was performed with anti-CD95 and anti-PLCγ1 mAbs. Nuclei were stained in blue (DAPI). Red dots were observed when the distance between anti-CD95 and anti-PLCγ1 mAbs was close (≈16 nm). *Right panels*: Red dots were counted in 200 cells taken from different fields. Data represent means ± SD of three independent experiments. (C) Schematic diagram of CD95 constructs. (D) CEM-IRC cells expressing GFP alone or the GFP-fused CD95 constructs shown in (C) were loaded with the Ca^2+^ probe, Fluo2-AM (1 μM). Cells were stimulated with cl-CD95L (100 ng/mL; arrow), and the intracellular calcium concentration ([Ca^2+^]_i_) was monitored. Data are given as means ± SD of three experiments performed independently on n = 20 cells. (E) HEK cells transfected with the indicated constructs were stimulated with CD95L (100 ng/mL) for indicated times. The CD95 protein complex was immunoprecipitated from cell lysates and subjected to immunoblotting, as indicated. Total lysates served as controls. The column marked “B” indicates treatment with beads alone. Data are representative of three independent experiments. (F) Activated PBLs were pre-incubated for 1 hr with TAT-control or TAT-CID (10 μM) and stimulated with cl-CD95L (100 ng/mL) for the indicated times. The CD95 protein complex was immunoprecipitated from cell lysates and subjected to immunoblotting, as indicated. Total lysates served as controls. The column marked “B” indicates a treatment with beads alone. Data are representative of three independent experiments. (G) Activated PBLs from healthy donors were loaded with FuraPE3-AM (1 μM) and pre-treated for 1 hr with TAT-control or TAT-CID (10 μM). Cells were stimulated with cl-CD95L (100 ng/mL; arrow). Data represent means ± SD.

We previously showed that engagement of CD95 evoked a Ca^2+^ response in activated T lymphocytes, resulting in transient inhibition of cellular apoptosis (Khadra et al., 2011) and cell migration (Malleter et al., 2013, Tauzin et al., 2011). Because of the instrumental role of the CD95-mediated apoptotic signal in anti-infectious and anti-tumor responses, we assumed that inhibiting CD95 non-apoptotic responses while conserving the apoptotic signal could be an attractive therapeutic option for preventing Th17 recruitment in inflamed organs without altering immune surveillance. We then wondered whether it was possible to selectively inhibit the CD95-mediated Ca^2+^ response and whether this blockade could inhibit cell migration without blocking the apoptotic signal. Human T cells exposed to cl-CD95L rapidly formed a transient molecular complex containing phospholipase Cγ1 (PLCγ1) (Figure 4A). Using a proximity ligation assay (PLA), we confirmed that this interaction was selective for CD95 and occurred in a transient and rapid manner (Figure S3A). CD95-PLCγ1 interaction was not detected in CEM-IRC cells, which express a faint amount of endogenous CD95 (Bénéteau et al., 2008), and the peak of interaction in parent CEM T cells was reached 5 min after addition of cl-CD95L (Figure S3A). Moreover, the lack of PLCγ1 in T cells resulted in a loss of CD95-mediated Ca^2+^ response (Figure S3B). PLA experiments also revealed that CD95 rapidly recruited PLCγ1 in Th17 cells exposed to cl-CD95L (Figure 4B). We next investigated whether the predominant DISC components, FADD and caspase-8, might contribute to CD95-mediated Ca^2+^ signaling. FADD- and caspase-8-deficient Jurkat T cells (Juo et al., 1998, Juo et al., 1999) exposed to the cytotoxic CD95L showed no apoptotic signaling (Figure S3C), but the CD95-mediated Ca^2+^ signaling pathway remained unaffected in these cells (Figure S3D), suggesting that PLCγ1 activation occurs independently of CD95-DISC and cell death signaling. To further investigate whether the death domain (DD) of CD95 was involved in the Ca^2+^ response, we generated CD95 constructs devoid of the entire intracellular domain of CD95 (CD95^1–175^), the DD (CD95^1–210^), or the last 15 aa involved in FAP-1 protein tyrosine phosphatase recruitment (CD95^1–303^) (Sato et al., 1995) (Figure 4C). These constructs were expressed in the CEM-IRC T cell line, which was selected for its low CD95 expression (Figure S3E).

CEM-IRC showed minimal cell death in response to a multimeric (dodecamer) and cytotoxic CD95L (IgCD95L, Figure S3F); however, expression of CD95^1–303^ or wild-type CD95 restored cell death levels to those observed for parental CEM cells (Figure S3F). In contrast, introduction of CD95^1–175^ or CD95^1–210^ failed to induce apoptosis, and as previously observed, these constructs behaved as dominant-negative receptors (Figure S3F) (Siegel et al., 2000). Furthermore, reconstituting CEM-IRC cells with wild-type CD95 or CD95^1–303^ restored CD95-mediated Ca^2+^ signaling (Figure 4D).

Whereas the loss of the DD from the CD95^1–210^ construct prevented apoptotic signaling, CD95-DD deletion did not affect induction of Ca^2+^ signaling (Figure 4D). Given that a CD95 construct devoid of the entire intracellular region (CD95^1–175^) failed to evoke a Ca^2+^ response, we concluded that Ca^2+^ signaling was triggered by CD95 amino acids 175–210.

We next examined whether CD95^1–210^ was capable of recruiting PLCγ1. To this end, we transiently co-transfected HEK cells with GFP-fused CD95 constructs and wild-type PLCγ1. Although the transfected cells expressed similar quantities of CD95-GFP chimeric constructs at the cell surface (Figure S3G), PLCγ1 was absent only from the CD95^1–175^ immunoprecipitate (Figure 4E). To demonstrate that amino acid residues 175–210 of CD95 contribute to PLCγ1 recruitment, we generated a construct comprising CD95^175–210^ (termed the calcium-inducing domain [CID]) fused to the fluorescent protein mCherry. Unlike mCherry alone, CD95^175–210^-mCherry interacted with PLCγ1 and inhibited its recruitment to CD95 (Figure S3H), suggesting that interference with this juxtamembrane domain might prevent CD95-mediated Ca^2+^ signaling.

To confirm this hypothesis, we synthesized a cell-penetrating peptide, TAT-CID, by linking CID to the nine-amino-acid HIV-TAT sequence (Figure S4A), which serves as a carrier for protein translocation across the plasma membrane (Vivès et al., 1997). Pre-incubation of activated peripheral blood lymphocytes (PBLs) with TAT-CID impaired the recruitment of PLCγ1 (Figure 4F) and abolished the induction of CD95-mediated Ca^2+^ signaling (Figure 4G). Similarly, pre-incubation of CEM cells with TAT-CID inhibited PLCγ1 binding to CD95 (Figure S4B) and abrogated the CD95-induced Ca^2+^ response in Jurkat and CEM T cell lines (Figure S4C). TAT-CID also reduced Akt phosphorylation at serine 473 (a hallmark of PI3K signaling activation) in cl-CD95L-exposed PBLs (Figure S4D), confirming a role of calcium in the modulation of the PI3K signaling pathway (Malleter et al., 2013). Nevertheless, although TAT-CID impeded CD95-mediated Ca^2+^ and PI3K signaling, the peptide did not affect the apoptotic signaling pathway (Figure S4E). Hence, we mapped a domain in the CD95 receptor, namely CID, which recruits PLCγ1 and elicits Ca^2+^ responses.

### CID Interacts with the SH3 Domain of PLCγ1

**Figure 5:**
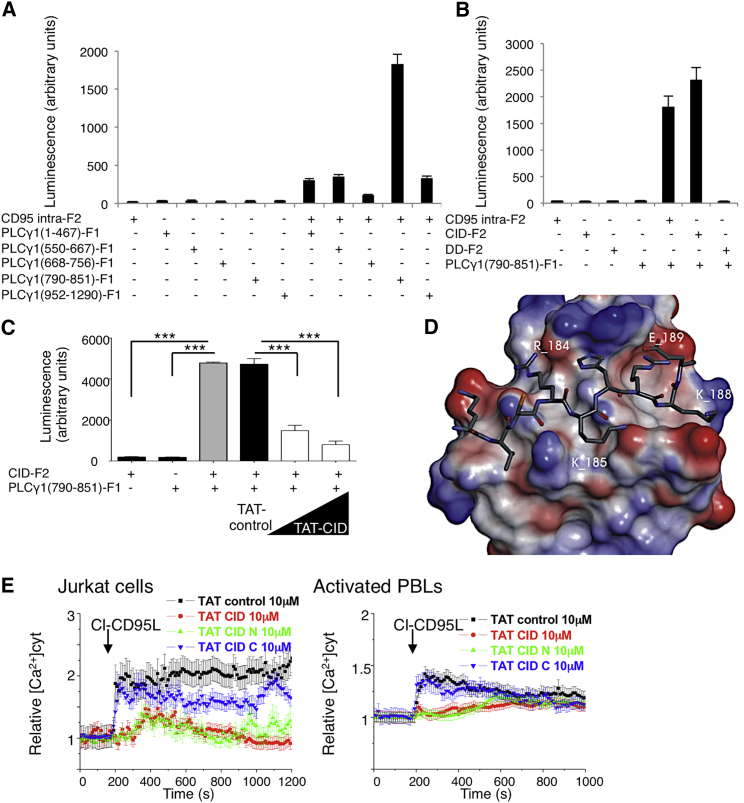
CID Interacts with the SH3 Domain of PLCγ1 (A) The intracellular region of CD95 (amino acids 175–319) fused to F2 was co-transfected into HEK cells with the indicated domains of PLCγ1 fused to F1. Refolding of the luciferase and reconstitution of enzyme activity revealed protein/protein interactions. Data represent means ± SD of three independent experiments. (B) The PLCγ1-SH3 domain (amino acids 790–851) fused to F1 was co-transfected into HEK cells with the indicated regions of CD95 fused to F2, and luminescence was assessed. Data represent means ± SD of three independent experiments. (C) CD95-CID-F2 and PLCγ1-SH3-F1 were co-transfected into HEK cells. Transfected cells were pre-incubated for 1 hr with TAT-control (50 μM) or TAT-CID (50 and 10 μM), and luminescence was measured. (D) Predicted interaction between CID and PLCγ1. Within CD95-CID, TCRKHRK is the amino acid sequence that is optimal for interaction with PLCγ1-SH3. (E) Indicated cells were pre-incubated for 1 hr with TAT-control, TAT-CID, TAT-CID-N, or TAT-CID-C (10 μM) and then stimulated with cl-CD95L (100 ng/mL). [Ca^2+^]_CYT_ was assessed in FuraPE3-AM (1 μM)-loaded cells. Data represent means ± the SD of three independent experiments.

To address whether CD95 directly interacts with PLCγ1, we performed a Renilla luciferase-based protein fragment complementation assay (RLuc-PCA) (Stefan et al., 2007). The Renilla luciferase enzyme was divided into two fragments. Each fragment (F1 and F2) was fused to various CD95 and PLCγ1 domains and co-transfected in HEK cells (Figures S5A–S5D). The PLCγ1-SH3 interacted with both the whole CD95 intracellular region and CD95-CID, but it failed to reconstitute enzyme activity when combined with CD95-DD (Figures 5A and 5B). To strengthen this result, we evaluated whether the cell-penetrating TAT-CID peptide could inhibit the interaction of PLCγ1-SH3 with CD95-CID. Although TAT-control peptide did not alter luciferase activity in cells co-expressing PLCγ1-SH3-F1 and CD95-CID-F2, TAT-CID efficiently blocked this activity in a dose-dependent manner (Figure 5C), supporting the hypothesis that CD95 directly associates with PLCγ1 through the CD95-CID domain. Intriguingly, SH3 domains primary bind to peptides containing a consensus PxxP sequence that is not present in CID; however, many examples of unconventional SH3-binding peptides have previously been described (Saksela and Permi, 2012).

To understand how CD95 CID might interact with PLCγ1-SH3, we undertook two computational peptide screenings. First, we performed homology modeling by using the crystal structure of PLCγ1-SH3 in association with the SLP-76 heptapeptide complex (PDB:1YWO) (Deng et al., 2005). This structure served as a template, and the SLP-76 peptide was replaced by each heptapeptide combination present in CD95-CID. Second, we took a protein-peptide docking approach by using each aforementioned CID heptapeptide alternatively docked within the PLCγ1-SH3 domain. Both methods predicted that amino acids 182–188 (TCRKHRK) would have the highest affinity for PLCγ1-SH3, and the calculated binding energy of the complex consisting of PLCγ1-SH3 and TCRKHRK was similar to that of the PLCγ1-SH3 interaction with SLP-76 (Figure 5D and Table S3). To prove that the TCRKHRK amino acid sequence corresponded to the minimal domain interacting with PLCγ1, we first used RLuc-PCA and performed an alanine-scanning experiment. This confirmed that amino acid residues R184, K185, and K188 were instrumental in the interaction of CD95 with PLCγ1 (Figure S5E). In agreement with this cell-based assay, our computer model identified an interaction between R184 guanidine and SH3-PLCγ1 through a hydrogen-bond network involving the side chains of Y845 and Q805 and the backbone of K803. K185 amine function forms hydrogen bonds, in an intramolecular manner, with the H186 and G826 backbones of SH3-PLCγ1. A salt bridge occurred between K188 and E825. Furthermore, the positioning of K188 in the SH3 cavity required the displacement of a water molecule (H_2_O105 observed in the crystal structure), an entropically favorable process. For this solvent-exposed interface, the methylene chain of these basic residues forms extensive van der Waals contacts with the SH3 hydrophobic shallow pockets. Overall, these observations indicated that even if the CID sequence is not related to a typical class II ligand of SH3, it complies with its properties in terms of van der Waals interactions (Deng et al., 2005). Of note, RLuc-PCA also revealed that glutamic acid 189, localized outside the minimal TAT-CID domain, participated in the binding of CD95 with PLCγ1 (Figure S5E). This amino acid may play a role of “compass residue” evoked for typical proline-rich SH3 ligands (Musacchio, 2002). Indeed, the canonical PxxP motif-containing peptides could be docked in two opposite orientations regarding the relative positioning of a positively charged residue (+xxPxxP or xPxxPx+) interacting with a negatively charged cleft on the SH3 surface (Saksela and Permi, 2012). Similarly to this basic amino acid in the PXXP motif, E189 could contribute to the positioning of the minimal CD95-CID peptide in the PLCγ1 SH3 domain.

Second, we generated two constructs, CD95 in which R184 and K185 were replaced by alanine (double mutant, or DM) and CD95 in which R184, K185, and K188 were replaced by alanine (triple mutant, or TM). These two constructs and wild-type CD95 were transfected into HEK cells, and the CD95-mediated Ca^2+^ response was evaluated in these cells. HEK cells express endogenous wild-type CD95, and therefore we expected expression of our constructs to create heterotrimeric complexes consisting of wild-type and mutated CD95, which could inhibit PLCγ1 recruitment in a dominant-negative fashion. Both double and triple mutants abrogated the CD95-mediated Ca^2+^ signaling pathway (Figure S5F). For each amino acid, we also generated single mutatants. Strikingly, when expressed in HEK cells, these mutants did not alter the CD95-mediated Ca^2+^ signaling pathway (data not shown), suggesting that unlike double and triple mutations, a single mutation inside the minimal CID did not disrupt PLCγ1 recruitment. Because CID corresponds to a disorganized domain, we surmised that increasing PLCγ1 binding affinity requires a larger zone of contact and that elimination of only one amino acid might not be sufficient to impair this recruitment in a heterotrimeric complex containing wild-type CD95. Overall, these findings indicate that the CD95-CID amino-terminal region corresponds to an unconventional SH3-binding peptide.

To confirm our in silico result, we synthesized two cell-penetrating peptides consisting of the amino- and carboxyl-terminal regions of CD95 CID (CID-N, corresponding to aa 175–192, and CID-C, corresponding to aa 193–210) (Figure S5G). We then evaluated their effects on the CD95-mediated Ca^2+^ response (Figure 5E), and showed that only CID-N abrogated CD95-mediated Ca^2+^ signaling in Jurkat cells and activated PBLs (Figure 5E). Taken together, these findings demonstrated that the juxtamembrane region of CD95 directly interacts with PLCγ1-SH3 to evoke Ca^2+^ signaling.

Lastly, we examined whether the inhibitory actions of TAT-CID were selective for CD95-mediated Ca^2+^ signaling. Although T cell receptor (TCR) stimulation led to a PLCγ1-dependent Ca^2+^ response in Jurkat cells (Figure S5H), pre-treatment with the TAT-CID peptide had no such effect (Figure S5I). Similarly, TAT-CID did not influence the PLCβ-driven Ca^2+^ response stimulated by carbachol, a cholinergic agonist that activates G-protein-coupled receptors to release calcium (Figure S5J). Therefore, TAT-CID selectively inhibits CD95-mediated Ca^2+^ signaling.

### Inhibition of CD95-Mediated Ca^2+^ Signaling Prevents Th17 Cell Transmigration In Vivo

**Figure 6:**
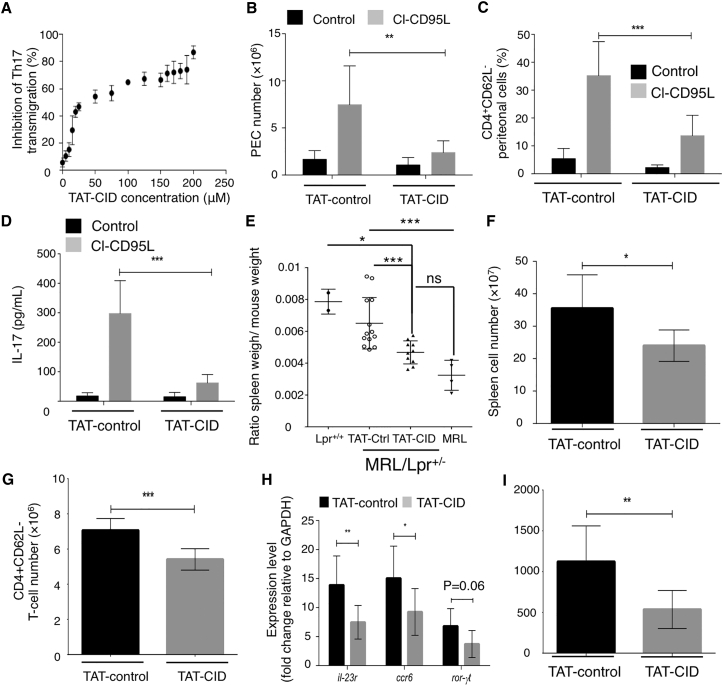
TAT-CID Alters Immunological Parameters in Lupus-Prone Mice (A) Transmigration of mouse Th17 cells was monitored with the indicated concentrations of TAT-CID. (B–D) C57BL/6 mice were injected with TAT-control or TAT-CID (40 mg/kg) 2 hr prior to intraperitoneal injection of cl-CD95L (200 ng) or vehicle. Animals were examined 24 hr after cl-CD95L injection. (B) Total cell counts in the peritoneal cavity are shown. (C) PECs were subjected to magnetic bead separation to identify the percentage of infiltrating CD4^+^CD62L^−^ (activated) T cells. (D) IL-17A concentrations in the peritoneal cavity were measured via ELISA (two-way ANOVA). Data in (B)–(D) represent two independent experiments performed with six mice/group. Data are means ± SEM. (E–I) MRL.*Fas*^*lpr/+*^ mice received either TAT-CID or TAT-control for 5 weeks. (E) Upon completion of the experimental protocol, ratios of spleen weight to body weight of individual animals were measured and compared to those of age-matched MRL and homozygous MRL.*Fas*^*lpr/lpr*^ mice. (F) Total cell number in the spleen is shown. (G) Cellular composition of the spleen was determined in regard to the number of CD4^+^CD62L^−^ T cells. (H) mRNA expression levels of *il-23r, ccr6*, and *ror-γt* in cells from (C). (I) Isolated T cells from (G) were re-stimulated with anti-CD3 mAb for 72 hr. IL-17A was then quantified by ELISA (unpaired Student’s t test).

To assess the potential of TAT-CID as a therapeutic agent for SLE patients, we first examined its effect on Th17 cell transmigration. Figure 6A shows that TAT-human CID (TAT-hCID) dose dependently inhibited CD95-mediated endothelial transmigration of human Th17 cells. Alignment of the human and mouse CD95 protein sequences highlighted differences in the CID region (21.2% sequence identity) (Figure S6A). We reconstituted human CEM-IRC cells with mouse CD95 (Figure S6B). CD95-mediated apoptotic signaling was restored in the mouse CD95-expressing cells, but not in the parental CEM-IRC cells (Figure S6B). TAT-mouse CID (TAT-mCID), but not TAT-hCID, inhibited the mouse CD95-mediated Ca^2+^ response (Figures S6C and S6D). Similarly, TAT-mCID also inhibited the CD95-mediated Ca^2+^ response in mouse T lymphocytes (Figure S6E), indicating that despite differences in the human and mouse CD95-CID sequences, both domains trigger Ca^2+^ signaling.

We next injected C57Bl/6 mice with TAT-control or TAT-mCID prior to the intraperitoneal injection of cl-CD95L and then determined the number of T cells infiltrating the peritoneal cavity 24 hr later. TAT-mCID treatment reduced the CD95-mediated accumulation of peritoneal exudate cells (PECs) (Figure 6B) and CD4^+^ lymphocytes (Figure 6C). In agreement with the data shown in Figure 3, cl-CD95L injection increased IL-17 levels in the peritoneal cavity, but the increase was overturned by TAT-mCID pre-treatment (Figure 6D). These findings indicated that TAT-CID inhibits cl-CD95L-mediated recruitment of IL-17-secreting CD4^+^ T cells in vivo.

### TAT-CID Alleviates Clinical Outcomes in Lupus-Prone MRL.*Fas*^*lpr/+*^ Mice

Patients suffering from ALPS type Ia exhibit CD95 mutations that cause SLE-like autoimmunity (Drappa et al., 1996, Fisher et al., 1995, Rieux-Laucat et al., 1995). Because of the insertion of a retrotransposon into intron 2 of the CD95 gene, heterozygous MRL.*Fas*^*lpr/+*^ mice express reduced levels of CD95 and develop lupus (Adachi et al., 1993). T cells from both ALPS type Ia patients and MRL.*Fas*^*lpr/+*^ mice show loss of sensitivity to CD95-mediated apoptosis but retain normal activation of non-apoptotic signaling pathways (Legembre et al., 2004). We asked whether the implementation of CD95-mediated non-apoptotic signaling pathways in lupus-prone mice contributed to symptom severity. Because TAT-CID inhibited CD95-mediated Ca^2+^ response without affecting apoptotic signaling (Figures S4C–S4E), this peptide allowed us to address this question.

TAT-mCID and TAT-control peptides were administered to MRL.*Fas*^*lpr/+*^ heterozygote mice. After completion of the trial, animals were sacrificed, revealing an alleviation of splenomegaly in TAT-CID-treated mice relative to controls (Figure 6E), without any negative effect on whole-body weight (Figure S6F). Likewise, TAT-CID significantly reduced the weights of the inflamed kidneys and the mesenteric lymph nodes (Figure S6G). Examination of the cellular composition of the spleen in MRL.*Fas*^*lpr/+*^ mice revealed a significant decrease in total spleen cell number (Figure 6F) and activated CD4^+^ T cells (Figure 6G), but not B cells (Figure S6H). Additionally, TAT-CID significantly decreased Th17 cell infiltration in the spleen of TAT-CID- versus TAT-control-treated mice, as indicated by reduced expression levels of *Ccr6* and *Il23r*, and to a lesser extent *Rorc*, three key molecular markers of Th17 cells (Figure 6H). Re-stimulation of spleen-infiltrating CD4^+^ T cells confirmed that the immune cells failed to produce IL-17A in TAT-CID-treated mice (Figure 6I).

**Figure 7:**
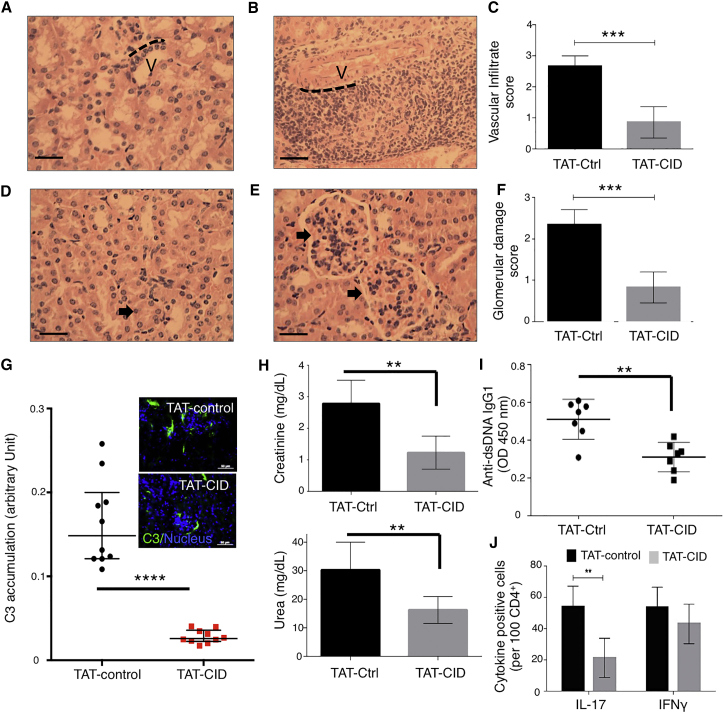
TAT-CID Alleviates Clinical Disease in Lupus-Prone Mice MRL.*Fas*^*lpr/+*^ mice received either TAT-CID or TAT-control twice weekly for 5 weeks. Kidneys were fixed, sectioned, and stained with hematoxylin and eosin. All images were captured at 20× ([B]; scale bar represents 250 μm) or 40× ([A, D, and E]; scale bar represents 150 μm) magnification. (A) Representative section of the vasculature (V) in a TAT-CID-treated mouse, with no obvious cellular infiltrate surrounding the border (delineated by the dashed black line). (B) Cellular infiltrate surrounding the vasculature (V) in a TAT-control-treated mouse, with multiple cell layers observed adjacent to the vasculature border (dashed black line). (C) Vascular infiltrate scores were calculated for the kidneys in TAT-CID- and TAT-control-treated mice. Data represent means ± SD (n = 8 mice/group; one-way ANOVA). (D) Kidney tissue showing normal glomeruli (arrow) and an adjacent tubule in TAT-CID-treated mice. (E) Kidney tissue showing pathological modifications (arrows) to the glomeruli in TAT-control-treated mice. (F) Glomerular-damage scores were calculated for each kidney in TAT-CID- and TAT-control-treated mice. Data represent means ± SD (n = 8 mice/group; ^∗∗∗^p < 0.001; Student’s t test). (G) C3 complement accumulation in kidneys of TAT-CID and TAT-control-treated MRL.*Fas*^*lpr/+*^ mice was assessed by microscopy. *Inset*: pictures of nucleus (DAPI) and C3 staining in mouse kidneys. (H) Serum creatinine (upper panel) and urea (lower panel) were measured. (I) Anti-dsDNA IgG1 amounts were determined by ELISA. Data represent means ± SD (n = 8 mice/group; Student’s t test). (J) Immunohistochemistry staining of IFN-γ or IL-17. Cytokine-positive cells were counted in treated kidneys.

Examination of the kidneys in MRL.*Fas*^*lpr/+*^ mice demonstrated that TAT-CID versus TAT-control decreased cell infiltration (Figures 7A–7C). The reduction in cellular infiltration in TAT-CID-treated MRL.*Fas*^*lpr/+*^ mice translated to a reduction of glomerulus damage (Figures 7D–7F). The number of cells infiltrating the glomeruli was significantly lower in TAT-CID-treated mice than in TAT-control mice, resulting in significant swelling and loss of shape of the glomeruli in these latter mice (Figure 7D versus Figure 7E). Moreover, improvement of the kidney architecture in TAT-CID-treated mice (Figure 7F) was associated with a decreased deposition of C3 activation fragments when these mice were compared to TAT-control mice (Figure 7G). Accordingly, organ function was restored in mice treated with repeated injections of TAT-CID as compared to TAT-control-treated mice with reduction of blood concentrations of creatinine and urea (Figure 7H). In parallel, serum concentrations of anti-dsDNA IgG1 were lower in TAT-CID mice than in TAT-control-treated MRL.*Fas*^*lpr/+*^ mice (Figure 7I). When kidneys of TAT-CID and TAT-control treated Lpr^+/−^ mice were analyzed, we found a lower number of CD4^+^IL17^+^ cells in the TAT-CID group than in the TAT-control-treated mice (Figure 7J). Although CD4^+^IFN-γ^+^ cell number tended to be lower in TAT-CID treated mice than in control mice, this effect was non-significant (Figure 7J). Treatment efficiency supported our prediction that CD95-induced non-apoptotic signaling pathways contribute to lupus severity and progression.

## Discussion

An initial study showing that activated T cells transmigrated in the presence of cl-CD95L through the implementation of PI3K and Ca^2+^ signaling pathways (Tauzin et al., 2011) raised the question of whether all T cells responded similarly to cl-CD95L and whether it was possible to selectively inhibit the CD95-mediated pro-inflammatory signaling pathway without affecting the apoptotic cues. Although the death domain of CD95 is instrumental in the induction of the PI3K signaling pathway (Tauzin et al., 2011), we here provided evidence that the Ca^2+^ response stemmed from a different CD95 region, which we identified and designated calcium inducing domain (CID).

CD95L^+^ blood vessels in skin of SLE patients were surrounded by infiltrating immune cells, suggesting that these structures could serve as “gateways” for inflammatory leukocytes and the ensuing recruitment of Th17 cells. By contrast, a recent study showed that CD95L expressed on endothelial cells functions to eliminate CD8^+^ T cells and in doing so, prevents effective anti-tumor immunity (Motz et al., 2014). Examination of the CD8^+^ T cell infiltrate surrounding CD95L^+^ blood vessels in SLE patients revealed no inverse correlation between CD95L expression and the number of infiltrating CD8^+^ T cells (Figures S7A and S7B). Given that CD95L exerts its chemoattractant activity only after cleavage by metalloproteases (Tauzin et al., 2011), the observed discrepancy between the amount of immune infiltrate surrounding CD95^+^ blood vessels in cancer subjects and SLE patients might be explained by the absence or presence of an as-yet-unidentified CD95L-processing metalloprotease.

The aggravation of SLE by cl-CD95L thus appears to involve a two-step mechanism. First, selective recruitment of Th17 and Th1 cells, conserved in both mouse and humans, occurs at the expense of Treg cell recruitment and might advance inflammation in damaged SLE organs. Second, Th17 cells exposed to cl-CD95L upregulate their expression of the PSGL-1 adhesion molecule. Not only does PSGL-1 promote tethering of lymphocytes to endothelial cells and subsequent rolling, but its high expression levels in T cells also provoke the secretion of effector cytokines (Baaten et al., 2013).

Of interest, the cellular balance between ceramide (apoptotic) and S1P (survival) is crucial for cell fate (Cuvillier et al., 1996), and although the interplay between CD95 and ceramide synthesis is well known and contributes to the induction of the cell-death program (Cifone et al., 1994), our findings provide evidence that a molecular link also exists between CD95 stimulation and activation of the S1P signaling pathway. Furthermore, activation of the S1P signaling pathway by cl-CD95L furnishes a molecular mechanism that can explain how T cells and, more specifically, Th17 cells migrate in presence of this naturally processed ligand. Based on our study and recent data from Yosef and colleagues, who used transcriptional profiling to reveal a role for CD95 in Th17 differentiation (Yosef et al., 2013), we conclude that CD95L/CD95 signals play a pivotal role in autoimmunity, and not only through a canonical apoptotic role.

We employed complementary approaches to reveal that CD95-CID directly interacts with the PLCγ1-SH3 domain to implement a Ca^2+^ signal. Linker for activation of T cells (LAT) is a critical adaptor molecule required for TCR-mediated Ca^2+^ responses. Upon TCR engagement, LAT recruits PLCγ1 via its SH2 domains (Zhang et al., 2000). CD95 and TCR therefore recruit PLCγ1 through different domains, and TAT-CID failed to inhibit TCR-mediated Ca^2+^ signaling in the current study. Moreover, blockade of CD95-mediated Ca^2+^ signaling by TAT-CID not only prevented selective recruitment of Th17 cells to the peritoneal cavity of cl-CD95-injected mice but also lessened clinical symptoms in heterozygous MRL.*Fas*^*lpr/+*^ mice. These observations implicate TAT-CID as an attractive therapeutic molecule with high selectivity toward the CD95-mediated Ca^2+^ signaling pathway.

Cl-CD95L induces the transient (within minutes) recruitment of PLCγ1. The CD95 domain encompassing amino acids 175–210 has never been crystallized, probably because this region corresponds to an intrinsically disordered region (IDR) that lacks a unique three-dimensional structure. We conducted several molecular-dynamics experiments to confirm that this peptide has very low propensity, if any, for folding (Figures S7C and S7D). Numerous examples of transient protein/protein interactions involving IDRs are now documented, indicating the importance of IDRs in allowing proteins to briefly associate with a large number of partners so as to dynamically modulate cell signaling (Cumberworth et al., 2013). This molecular feature is consistent with the CD95-CID-mediated induction of a rapid and transient Ca^2+^ response that promotes cell migration via the MISC formation.

A recent phase I/II clinical trial demonstrated that a decoy CD95 receptor, APG101, could impede CD95/CD95L interaction and benefited patients suffering from glioblastoma (Tuettenberg et al., 2012). Although APG101 might be of short-term therapeutic benefit to lupus patients, its inability to discriminate between the anti-tumor/infectious (i.e., apoptotic signaling) and pro-inflammatory actions of CD95 might lead to unexpected adverse events.

Because we have found that inhibiting the CD95-mediated Ca^2+^ response does not interfere with apoptotic signaling, we propose that selective blockade of CD95-mediated Ca^2+^ signaling might open new therapeutic avenues for SLE treatment in the future. Moreover, given the selective effect of cl-CD95L on Th17 cell recruitment, we also propose that downregulating Ca^2+^ signaling could lessen the inflammatory activity of Th17 cells in other chronic inflammatory disorders.

## Experimental Procedures

### Antibodies and Other Reagents

See Supplemental Experimental Procedures.

### Plasmids and Constructs

All constructs and primer pairs used are described in the Supplemental Information.

### T Cell Subset Isolation

Peripheral blood mononuclear cells (PBMCs) were isolated from buffy-coat by density gradient via lymphocyte separation medium (Eurobio). PBMCs were then subjected to selection with a cocktail of antibody-coated magnetic beads: CCR6^+^CXCR3^−^CD4^+^ cells (Th17 cells) were sorted with Human Th17 Enrichment kit (STEMCELL Technologies), and CD4^+^CD25^+^CD127^−^ Treg cells were isolated with MACS column (Miltenyi Biotec).

### Transcriptomic Analysis

Isolated Th17 and Treg cells from two healthy donors were treated with or without 100 ng/mL of cl-CD95L for 8 hr. Total RNA was extracted with the Nucleospin RNA XS kit (Macherey-Nagel), and quality was assessed with the RNA6000 nano chip (Agilent). For each condition, 9 ng of RNA was reverse transcribed with the Ovation PicoSL WTA System V2 (Nugen, Leek, The Netherlands). Fragmented cDNAs were hybridized to GeneChip Human Gene 2.0 ST microarrays (Affymetrix). Then chips were scanned on a GeneChip Scanner 3000 7G (Affymetrix). Raw data and quality-control metrics were generated from scanned images using the Expression Console software (Affymetrix).

Probes were mapped with Brainarray V20 CDF files (http://brainarray.mbni.med.umich.edu/) and normalized by robust multi-array averaging with R software. Statistical analyses were performed with Partek Genomics Suite; a p value ≤ 0,05 was considered significant. Pathway enrichment analyses were generated with Ingenuity Pathway Analysis (QIAGEN).

### MRL.*Fas*^*lpr/+*^ Mouse Treatment

MRL.*Fas*^*lpr/+*^ mice were obtained from the Jackson Laboratory and backcrossed onto a MRL background. Females (n = 8/group) were intraperitoneally administrated with either TAT-CID or TAT-Ctrl peptides (40 mg/kg) starting at 8 weeks of age, twice weekly for 5 weeks. After re-stimulation of CD4^+^ cell populations with anti-CD3, cell culture supernatants were dosed for IL-17A and IFN-γ by ELISA, and qPCR analysis of purified cell population was also performed as mentioned above. Kidneys were fixed into 4% PFA overnight prior to being placed in ethanol, sectioned, and stained. Scoring was conducted by an individual blind to the objective/treatments within the study as per (Kikawada et al., 2003). FITC-conjugated rabbit anti-C3 polyclonal antibody (Dako) and nuclei (DAPI, sigma-aldrich) staining was performed on frozen kidney sections, and results were analyzed with NIKON Ni-E (magnification × 200). C3 accumulation in kidneys was assessed by densitometric analysis of ten different fields via NIS-Elements AR Analysis software.

### Transendothelial Migration of Activated T Lymphocytes

Membranes (3 μm pore size) of a Boyden chamber were hydrated in sterile D-PBS (Millipore). Activated T-lymphocytes (10^6^) were added to the top chamber on a confluent monolayer of HUVEC in a low-serum (1%) medium. The bottom chamber was filled with low-serum (1%) medium in the presence or absence of 100 ng/ml of cl-CD95L. In experiments using human sera, 500 μl of serum from either healthy donors or SLE patients was added to the lower chamber. Cells were cultured for 24 hr at 37°C in a 5% CO_2_, humidified incubator. Transmigrated cells were counted in the lower reservoir by flow cytometry with a standard of 2.5 × 10^4^ fluorescent beads (Flow-count, Beckman Coulter).

## Author Contributions

A.P., D.S., M.L.G., M.M., R.V., L.M., A.P., A.F., F.J., E.F., T.D., A.M.V., P.A.B., R.J.F., and P.V. conducted the experiments. N.L. developed computer analyses. P.B., A.D., F.P., J.S., J.R., N.R., and C.C. provided reagents. R.J.F., P.V., and P.L. designed the experiments, analyzed data, and wrote the paper. P.L. supervised the project.

## Accession Numbers

Raw and normalized data are deposited to the GEO database under accession ID GSE78909.

## Supplemental Information

Document S1. Figures S1–S7 and Table S3

Table S1. Transcriptomic Analysis Isolated Th17 and Treg cells from two healthy donors were treated with or without 100 ng/mL cl-CD95L for 8 hr. Total RNA was extracted. Related to Figure 2.

Table S2. Transcriptomic Analysis Relative n-fold change in the quantity of transcripts significantly (p ≤ 0.05) and differentially expressed in Th17 and Treg cells either unstimulated or stimulated with cl-CD95L (100 ng/mL). Data for each experimental group (n = 2 per condition) are shown. Related to Figure 2.

Document S2. Article plus Supplemental Information
