# Supplementary material for: CD95-Mediated Calcium Signaling Promotes T Helper 17 Trafficking to Inflamed Organs in Lupus-Prone Mice
Source: Immunity. 2016 Jul 19;45(1):209–23. doi: 10.1016/j.immuni.2016.06.028 (PMC4961226; doi:10.1016/j.immuni.2016.06.028)
Supplement: Document S1. Figures S1–S7 and Table S3 [file mmc1.pdf]

Immunity, Volume *45*

Supplemental Information

CD95-Mediated Calcium Signaling

Promotes T Helper 17 Trafficking

To Inflamed Organs in Lupus-Prone Mice

Amanda Poissonnier, Doriane Sanséau, Matthieu Le Gallo, Marine Malleter, Nicolas Levoin, Roselyne Viel, Lucie Morere, Aubin Penna, Patrick Blanco, Alain Dupuy, Florence Poizeau, Alain Fautrel, Julien Seneschal, Florence Jouan, Jerome Ritz, Edouard Forcade, Nathalie Rioux, Cécile Contin, Thomas Ducret, Anne-Marie Vacher, Paul A. Barrow, Robin J. Flynn, Pierre Vacher, and Patrick Legembre

SUPPORTING FIGURE LEGENDS

Figure S1, related to Figures 1 and 2. Endothelial transmigration of Th17 CD4$^+$ T lymphocytes. A. The expression of CD95L or IL17 was analyzed by immunohistochemistry in inflamed skin from patients affected by lupus. Numbers represent different patients. **B.** HUVECs were incubated in the presence or absence of cl-CD95L (100 ng/mL) for 24 h. Next, the cells were detached using an EDTA-containing solution and stained with the indicated mAbs. The presence of the different proteins was detected by flow cytometry using a PE-conjugated secondary anti-mouse antibody. The histogram is representative of four independent experiments. **C.** The values for each indicated marker (*upper panels*: PSGL1 and *lower panel*: LFA-1) represent the mean fluorescence intensity (MFI) (± SD) measured by flow cytometry. For each Th subset, between 6 and 10 independent experiments were performed. **D.** Endothelial transmigration of human Th1, Th17, or Treg cells was assessed in the presence of neutralizing anti-E-selectin (10 μg/mL) antibody. Data were analyzed using two-way ANOVA. *P < 0.05 and ***P < 0.001. **E.** *Left panel:* Treg and Th17 cells were sorted from peripheral blood of healthy donors and the CD95 expression level at the surface of these cells was evaluated by flow cytometry. *Intermediate panel:* Comparison of CD95 Expression in different CD4$^+$T cell subsets: Naive CD4+ (CD45RA$^+$CD4$^+$), Memory CD4$^+$ (CD4$^+$CD45RA$^-$), Th1 (CD4$^+$CXCR3$^+$CCR6$^-$CD45RA$^-$), Th17 (CD4$^+$CXCR3$^-$CCR6$^+$CD45RA$^-$), Treg cells (CD4$^+$CD25^{high}FoxP3$^+$). *Right panel:* CD95 expression was normalized as follows: CD95 MFI in Th subset or Treg divided by CD95 in naive CD4+T. Wilcoxon paired analysis was performed in a set of 20 healthy donors. Histogram plots represent mean ±SD. **F.** Flow cytometry analyses demonstrating the efficiency of Treg (*left panels*) and Th17 cell (*right panels*) sorting from human peripheral blood. **G.** Schema of the S1P signaling pathway. In red, genes upregulated in Th17 cells stimulated with cl-CD95L.

Figure S2, related to Figure 3. Cl-CD95L is a chemo-attractant for Th17 cells *in vivo*. Mice received a single injection of cl-CD95L (200 ng) or vehicle prior to sacrifice 24 h later. All experiments were repeated three times. **A, B, C, D, E.** $CD4^{+}CD62L^{-}$ PECs were purified by AutoMACS separation, and RNA was prepared prior to real-time qPCR to assess expression levels of (A) IL-17A, (B) IL-23R, (C) CCR6, (D) IFN-γ, and (E) FoxP3 mRNA**.** Data represent means ± the SD (n = 6 mice per group; *P < 0.05, **P < 0.01, and ***P < 0.001; Student's t-test). GAPDH, glyceraldehyde 3-phosphate dehydrogenase.

Figure S3, related to Figure 4. Amino acid residues 175–210 of CD95 induce the Ca^{2+} signaling pathway. A. Parental CEM T-cells and their low CD95-expressing counterpart, namely CEM-IRC (for Ig-CD95L resistant cell) were stimulated with cl-CD95L (100 ng/mL) for indicated time points. PLA was performed using anti-CD95 and anti-PLCγ1 mAbs. Nuclei are stained in blue (DAPI). Red dots were observed when the distance between anti-CD95 and anti-PLCγ1 mAbs was close (≈16 nm). *Inset:* The expression level of CD95 was evaluated in Parental CEM and CEM-IRC cells by flow cytometry. **B.** Parental Jurkat T-cells and their PLC-γ1-deficient and PLC-γ1-reconstituted counterparts were loaded with the Ca^{2+} probe, FuraPE3-AM (1 μM), prior to stimulation with cl-CD95L (100 ng/mL; black arrow). Ratio images (F340/F380, R) were taken every 10 s and normalized against pre-stimulated values (R_0). Data represent the mean ± SD of R/R_0. *Inset:* PLCγ1-deficient Jurkat cells or their reconstituted counterparts were lysed, and the expression of PLCγ1 and CD95 was evaluated by immunoblotting. Tubulin was used as a loading control. **C.** Parental Jurkat cells (A3) and their counterparts lacking FADD or caspase-8 were treated with the indicated concentrations of cytotoxic IgCD95L for 20 h. Cell death was assessed using MTT assay. **D.** Indicated cells were loaded with the Ca^{2+} probe FuraPE3-AM (1 μM) and then stimulated

with cl-CD95L (100 ng/mL). Data were analyzed as described in B. *Inset:* Parental Jurkat cells (A3) or their counterparts lacking either FADD or caspase-8 were lysed, and the expression levels of CD95, FADD, and caspase-8 were examined by immunoblotting. **E.** The levels of CD95 expression at the cell surface of CEM-IRC clones reconstituted with the indicated CD95 constructs were evaluated by flow cytometry. Data are representative of three independent experiments. **F.** Cells described in E were treated with the indicated concentrations of IgCD95L for 20 h. Cell death was assessed using MTT assay. **G.** HEK cells were co-transfected with the GFP-fused CD95 constructs and wild-type PLCγ1. Twenty-four hours after transfection, the levels of CD95 expression were evaluated by flow cytometry. **H.** *Left panel:* HEK cells were co-transfected with PLCγ1 and mCherry or mCherry-CID. After 24 h, cells were lysed and PLCγ1 was immunoprecipitated. The immune complex was resolved by SDS-PAGE and subjected to immunoblot analysis as indicated. Total lysates were loaded as a control. *Right panel:* HEK cells were co-transfected with PLCγ1 and CID-mCherry or mCherry alone. After 24 h, cells were stimulated in the presence or absence of CD95L (100 ng/mL), and CD95 was immunoprecipitated. The immune complex was resolved by SDS-PAGE and immunoblotted as indicated. Total lysates were loaded as a control.

Figure S4, related to Figure 5. Cell-penetrating TAT-CID peptide inhibits the CD95-induced Ca^{2+} response without affecting the apoptotic signaling pathway. A. Protein sequences of TAT-CID and TAT-control. **B.** The leukemic T-cell line CEM was pre-incubated for 1 h with 10 μM of TAT-control or TAT-CID and then stimulated in the presence or absence of cl-CD95L (100 ng/mL) for the indicated times. Cells were lysed and CD95 was immunoprecipitated. The immune complex was resolved by SDS-PAGE and immunoblotted as indicated. Total lysates were loaded as a control. **C.** Jurkat and CEM cells were loaded with FuraPE3-AM (1 μM), pre-treated for 1 h with 10 μM TAT-control or TAT-

CID, and then stimulated with 100 ng/mL of cl-CD95L (black arrow). Ratio images were taken every 10 s and normalized against pre-stimulated values. **D.** Activated PBLs were pre-incubated for 1 h with 10 μM TAT-control or TAT-CID and then stimulated in the presence or absence of cl-CD95L (100 ng/mL) for the indicated times. Cells were then lysed, and 100 μg of protein was resolved by SDS-PAGE and immunoblotted as indicated. Total Akt served as a loading control. **E.** The indicated cells were pre-incubated for 1 h with a non-toxic dose of TAT-control or TAT-CID (10 μM), and cell death was assessed in an MTT assay.

Figure S5, related to Figure 5. CID-CD95 directly interacts with SH3-PLCγ1 domain. A. The Renilla luciferase enzyme was divided into amino-terminal and carboxy-terminal fragments (F1 and F2, respectively). **B.** Human PLCγ1 domains. PH, pleckstrin homology domain; cat, catalytic domain; and SH, src homology domain. **C.** HEK cells were transfected with indicated constructs. After 24 h, cells were lysed, and the expression levels of the different constructs were examined by immunoblotting using an anti-luciferase mAb recognizing the amino-terminal region of luciferase (MAB4410, Millipore, France). **D.** HEK cells were treated as in **C** and the expression levels of the indicated constructs were examined by immunoblotting using an anti-luciferase mAb recognizing the carboxy-terminal region of luciferase (MAB4400, Millipore). **E.** Each amino acid in CID sequence was replaced by alanine (alanine scanning) and all constructs were co-transfected with SH3-PLCγ1 in HEK cells. For each co-transfection, luminescence was assessed and a ratio was calculated as follows: (luminescence for mutated construct/ luminescence for wild type CID) × 100). For each condition, this value was next multiplied by the relative expression level of CID-F2 and SH3-PLCγ1-F1, evaluated by densitometric analysis of proteins as follows: ((densitometric value of mutated CID-F2 construct/ densitometric value of wild type CID-F2) × (densitometric value of co-transfected SH3-PLCγ1-F1/ densitometric value of SH3-PLCγ1-F1

co-transfected with wild type CID)). **F.** CID sequence of wild type CD95 (TCRKHRK) underwent double (TC**AA**HRK) or triple (TC**AA**HR**A**) mutations and these constructs were transiently transfected in HEK cells. Calcium signal was assessed using fluo2-AM (1 μM). Cells were stimulated with cl-CD95L (100 ng/mL; arrow), and the intracellular calcium concentration ($[Ca^{2+}]_i$) was monitored by measuring the F/F_0 ratio (relative cytoplasmic calcium concentration ($[Ca^{2+}]_{CYT}$)). **G.** Sequences of cell-penetrating peptides CID-N and CID-C. **H-I-J.** Indicated cells were loaded with the Ca^{2+} probe FuraPE3-AM (1 μM). Ratio images were taken every 10 s and normalized against pre-stimulated values**. H.** The anti-CD3 mAb OKT3 (1 μg/mL) evokes a PLCγ1-dependent Ca^{2+} response in Jurkat cells. **I.** Pre-incubation of Jurkat T-cells for 1 h with 10 μM TAT-CID or TAT-control had no effect on the CD3-induced Ca^{2+} response. **J.** The PLCβ-driven Ca^{2+} response induced by carbachol (10 μM) in Jurkat cells was not affected by TAT-CID pre-treatment (10 μM for 1 h).

Figure S6, related to Figure 6. A cell-penetrating murine CID inhibits the CD95-mediated Ca^{2+} response in murine T-cells. A. Mouse and human CD95 sequences were aligned using ClustalW2 (Larkin et al., 2007). **B.** *Inset:* CEM-IRC cells were reconstituted with murine CD95, a clone was selected, and its CD95 expression level was evaluated by flow cytometry. The sensitivity of murine CD95-expressing CEM-IRC to the cytotoxic CD95L, IgCD95L, was assessed using MTT assay. **C.** The cells were loaded with the Ca^{2+} probe FuraPE3-AM (1 μM). Ratio images were taken every 10 s and normalized against pre-stimulated values**.** Pre-incubation of murine CD95-expressing CEM-IRC T-cells with 10 μM TAT-mCID for 1 h inhibited the CD95-mediated Ca^{2+} response, whereas pre-incubation with TAT-hCID did not. **D.** The murine CID sequence fused to the cell-penetrating peptide TAT. **E.** Activated murine PBLs were loaded with the Ca^{2+} probe FuraPE3-AM (1 μM). Ratio images were taken every 10 s and normalized against pre-stimulated values**.** Pre-incubation of

activated murine PBLs with 10 μM TAT-mCID for 1 h inhibited the CD95-mediated Ca^{2+} response, whereas pre-incubation with TAT-hCID did not. **F-G-H.** 8-week old MRL/lpr+/- mice were administered with either TAT-CID or TAT-control peptide twice weekly for five weeks. Upon completion of the treatment, total body (**F**) and organ weight (**G**) were assessed. **H.** B-cell composition of the spleen was evaluated.

Figure S7, related to Figure 7. Infiltrating CD8 T-cells in skin lesions of lupus patients and computer modeling of CID. **A.** Expression of CD95L and CD8 was assessed by immunohistochemistry. The numbers represent the different patients. **B.** Densitometric analysis of the CD8 and CD95L staining depicted in **A** revealed no inverse correlation between CD8 and CD95L expression levels in skins of SLE patients. **C.** Observed backbone hydrogen bonds between pairs of residues (% as a function of the simulation time). Counting main chain hydrogen bonds during the 40 ns molecular dynamics analysis revealed that these interactions were rare. **D.** Dynamics of the peptide structures. RMSD and radius of gyration suggest that the system was stable, with no detectable compression of the peptide. This means that no stable secondary structures occurred. The presence of short structural elements was noticed from time-to-time, e.g., in the middle of the peptide at 5 ns and at the COOH-terminus at 13, 20, 30, and 37 ns. These folds correspond to discrete events, and the peptide did not adopt a stable 3D structure (the small α-helices have a short half-life and are quickly broken).

Reference

Larkin, M.A., Blackshields, G., Brown, N.P., Chenna, R., McGettigan, P.A., McWilliam, H., Valentin, F., Wallace, I.M., Wilm, A., Lopez, R., *et al.* (2007). Clustal W and Clustal X version 2.0. Bioinformatics *23*, 2947-2948.

Supplementary Figure 1

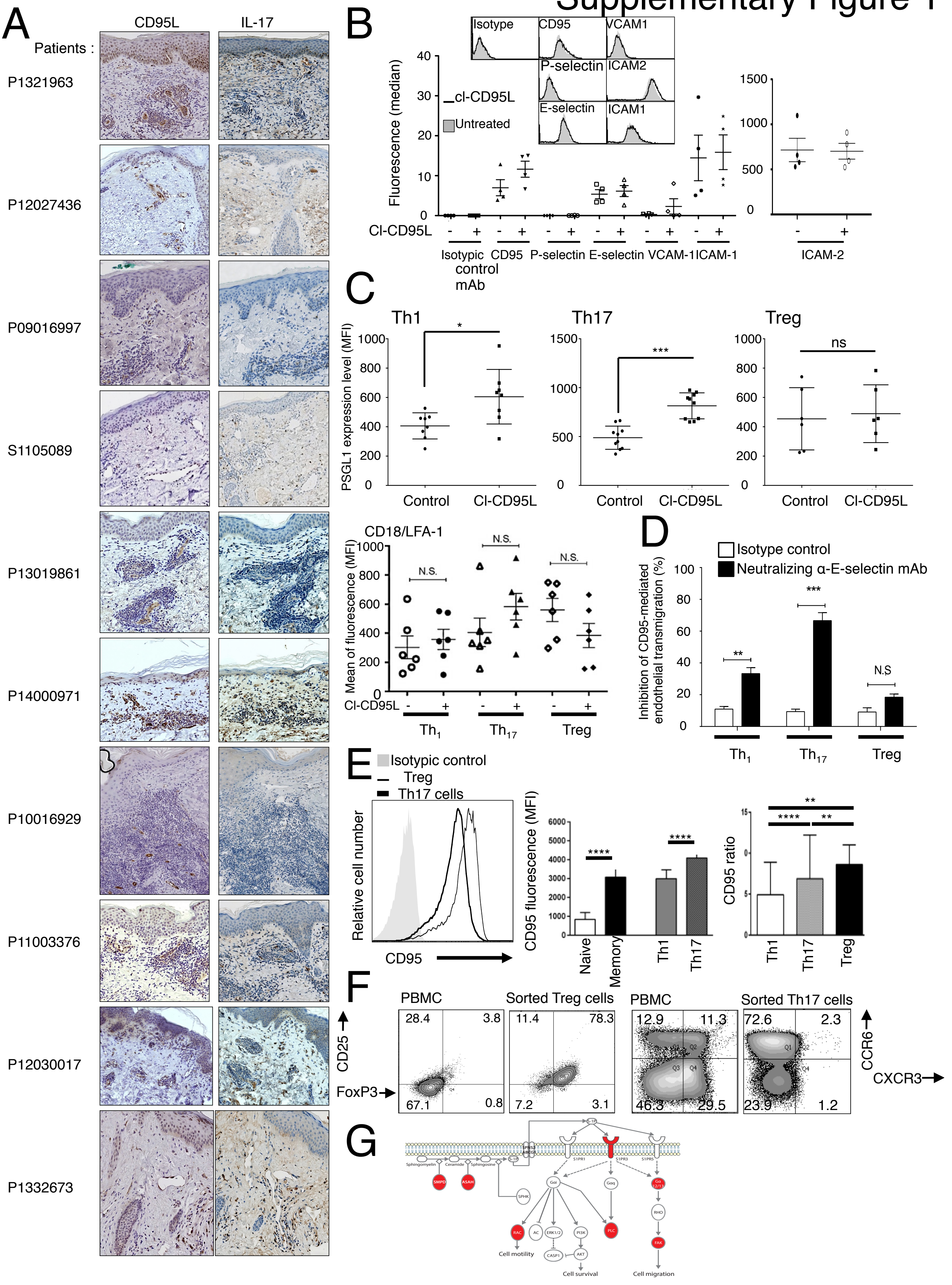

Supplementary Figure 2

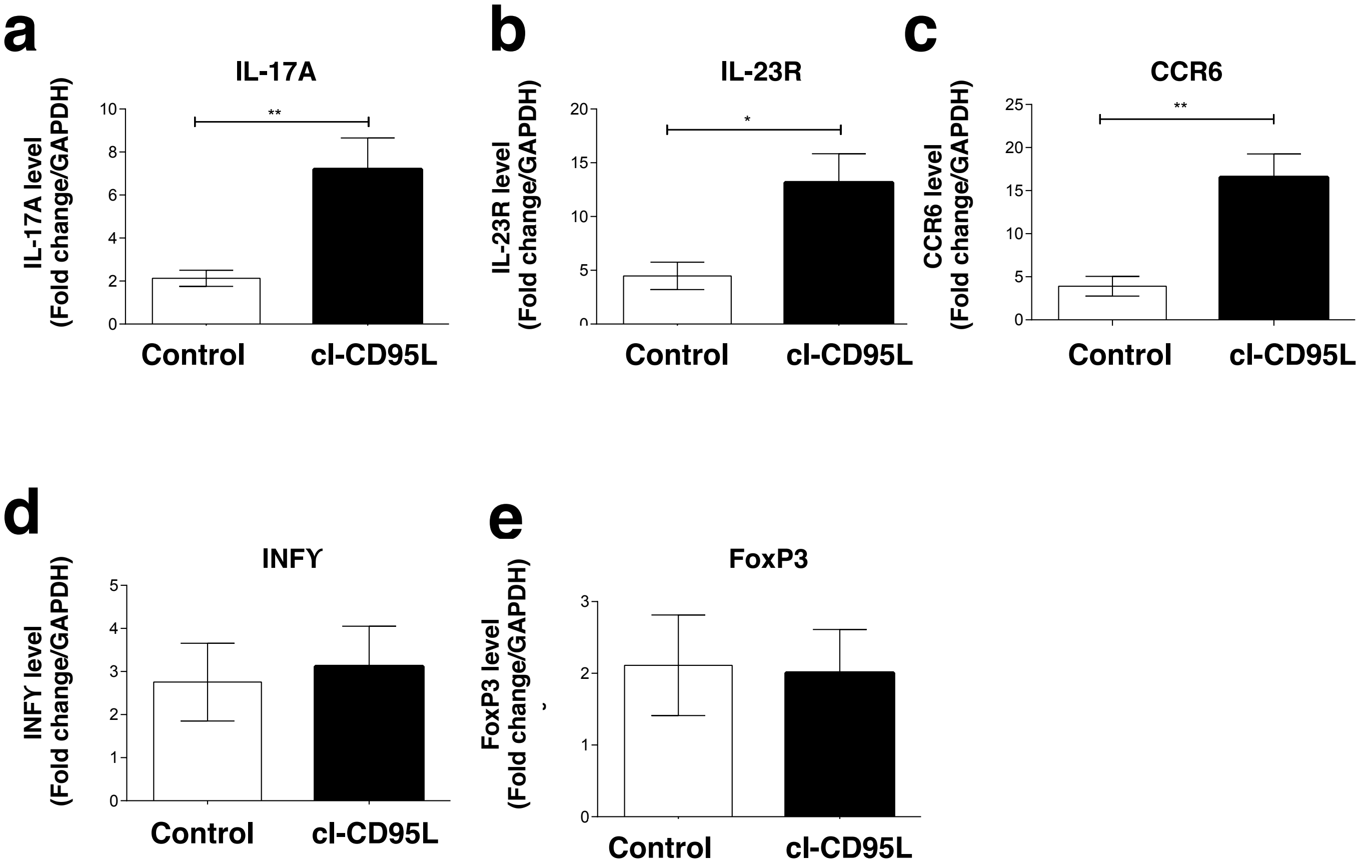

Supplementary Figure 3

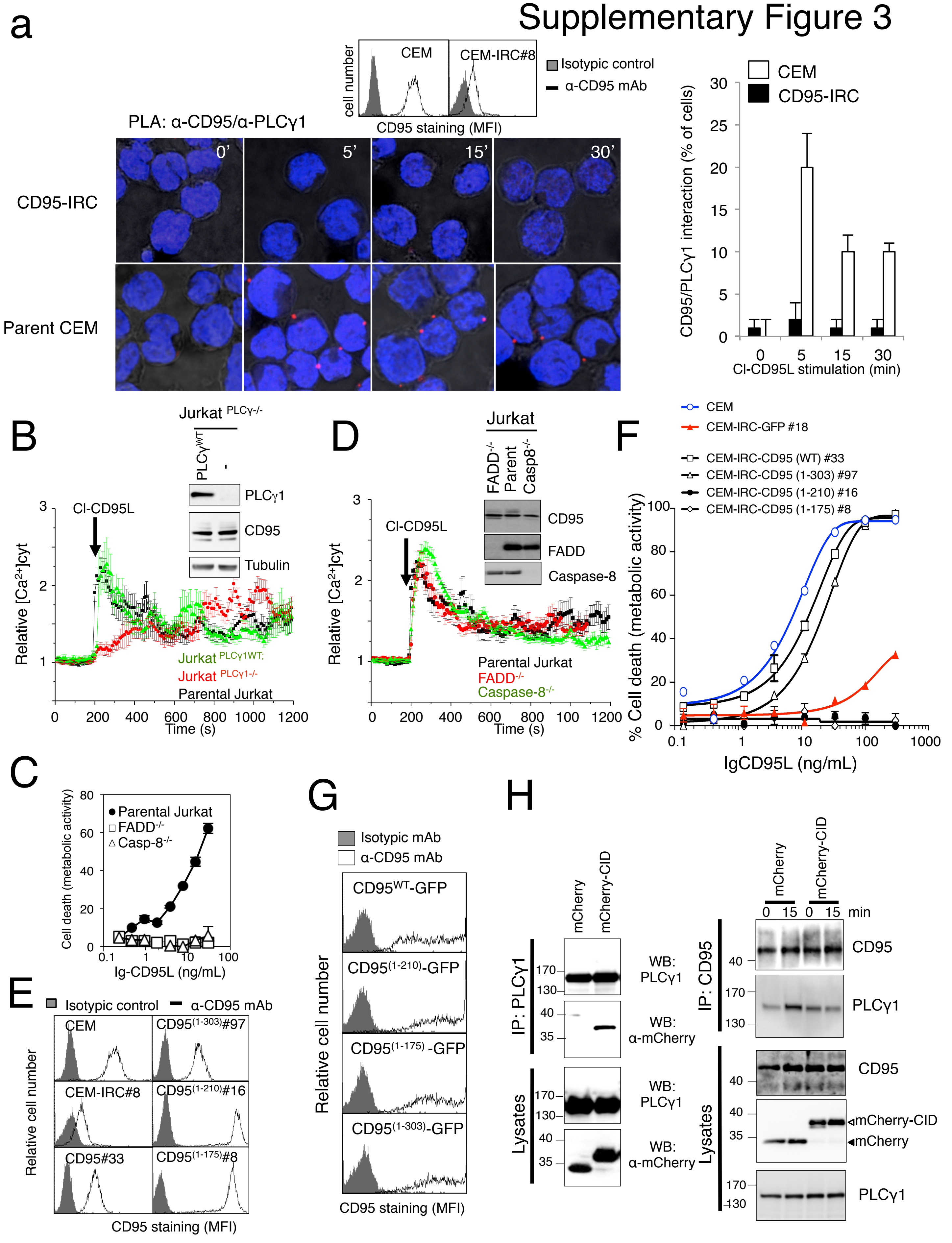

Supplementary Figure 4

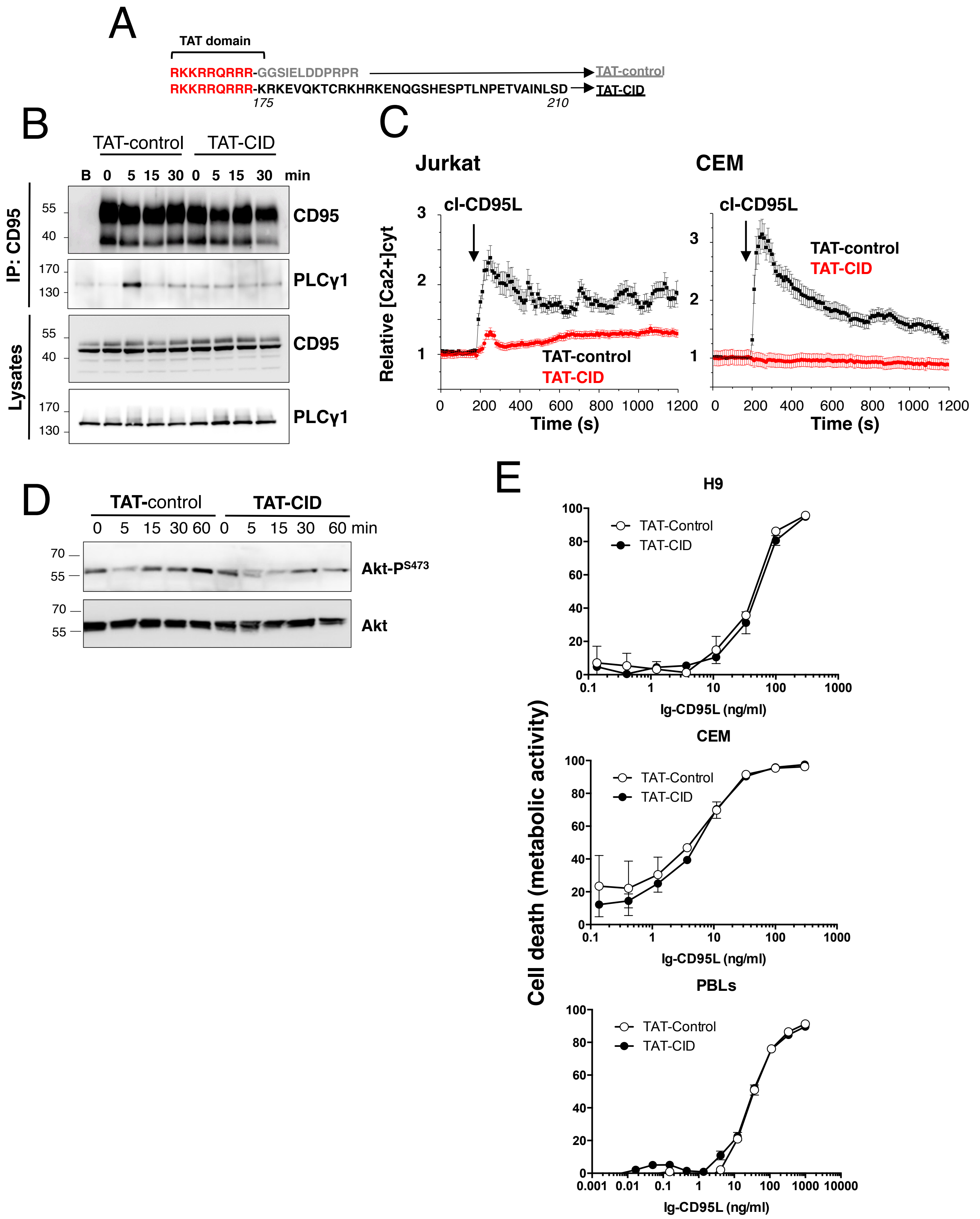

Supplementary Figure 5

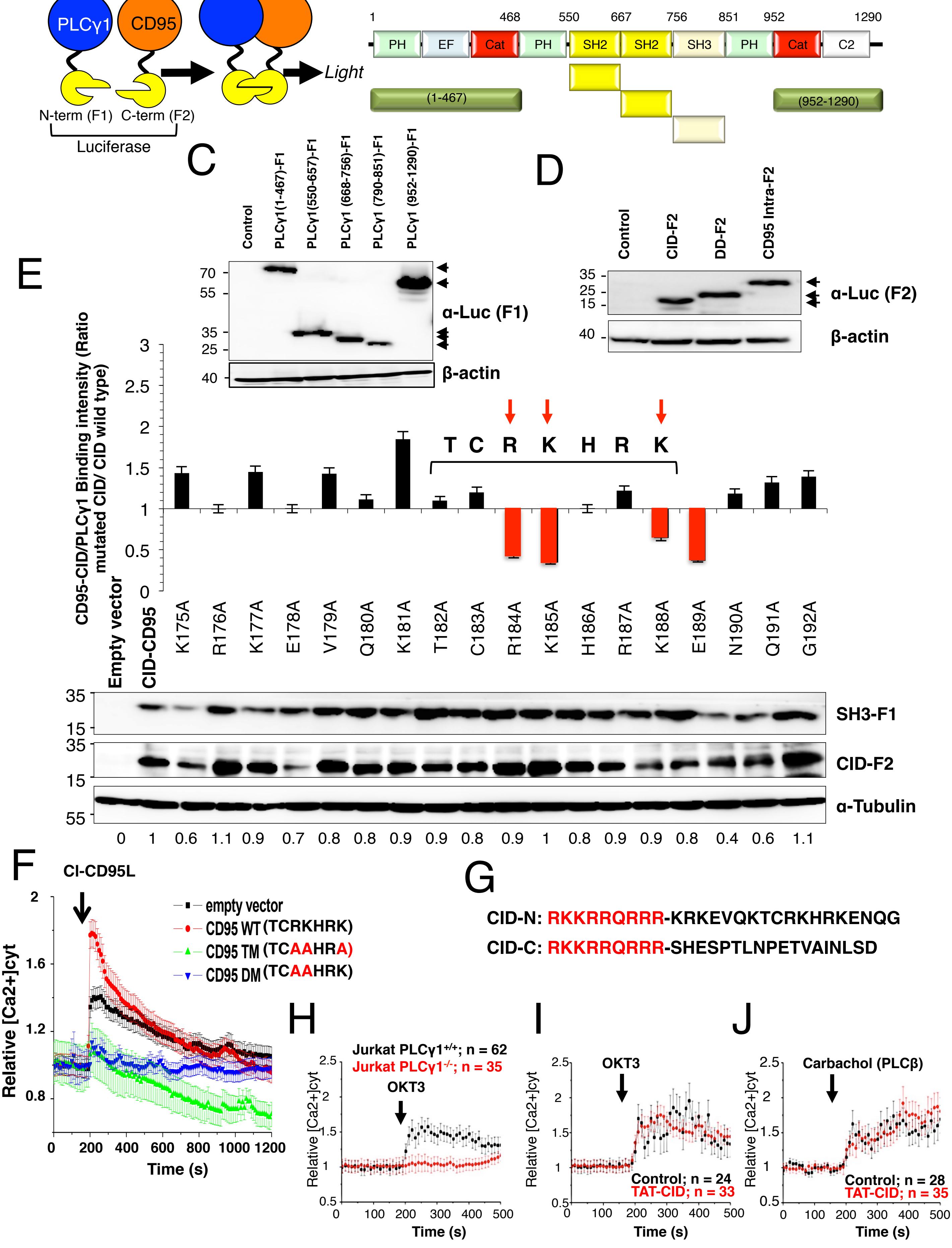

Supplementary Figure 6

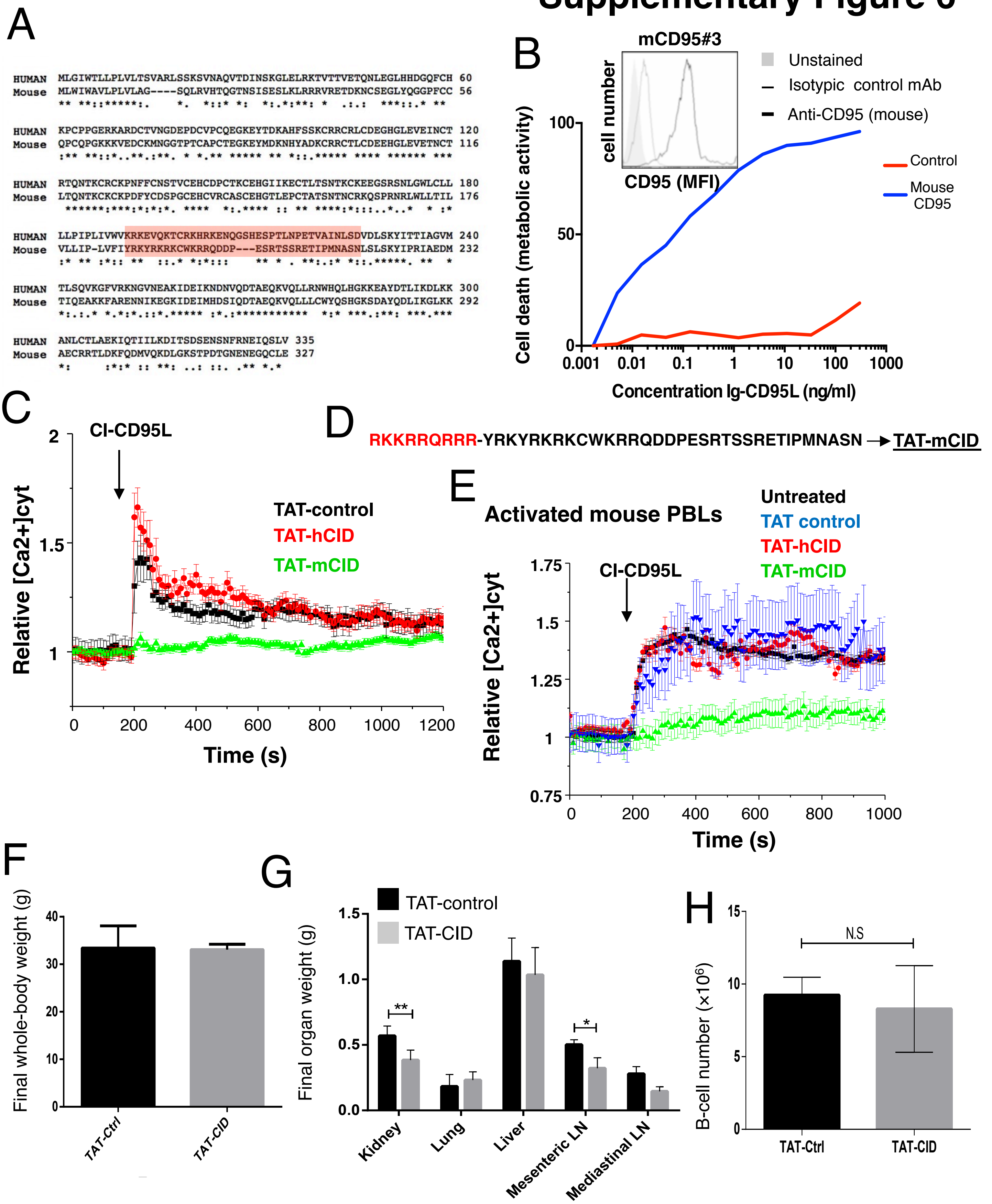

Supplementary Figure 7

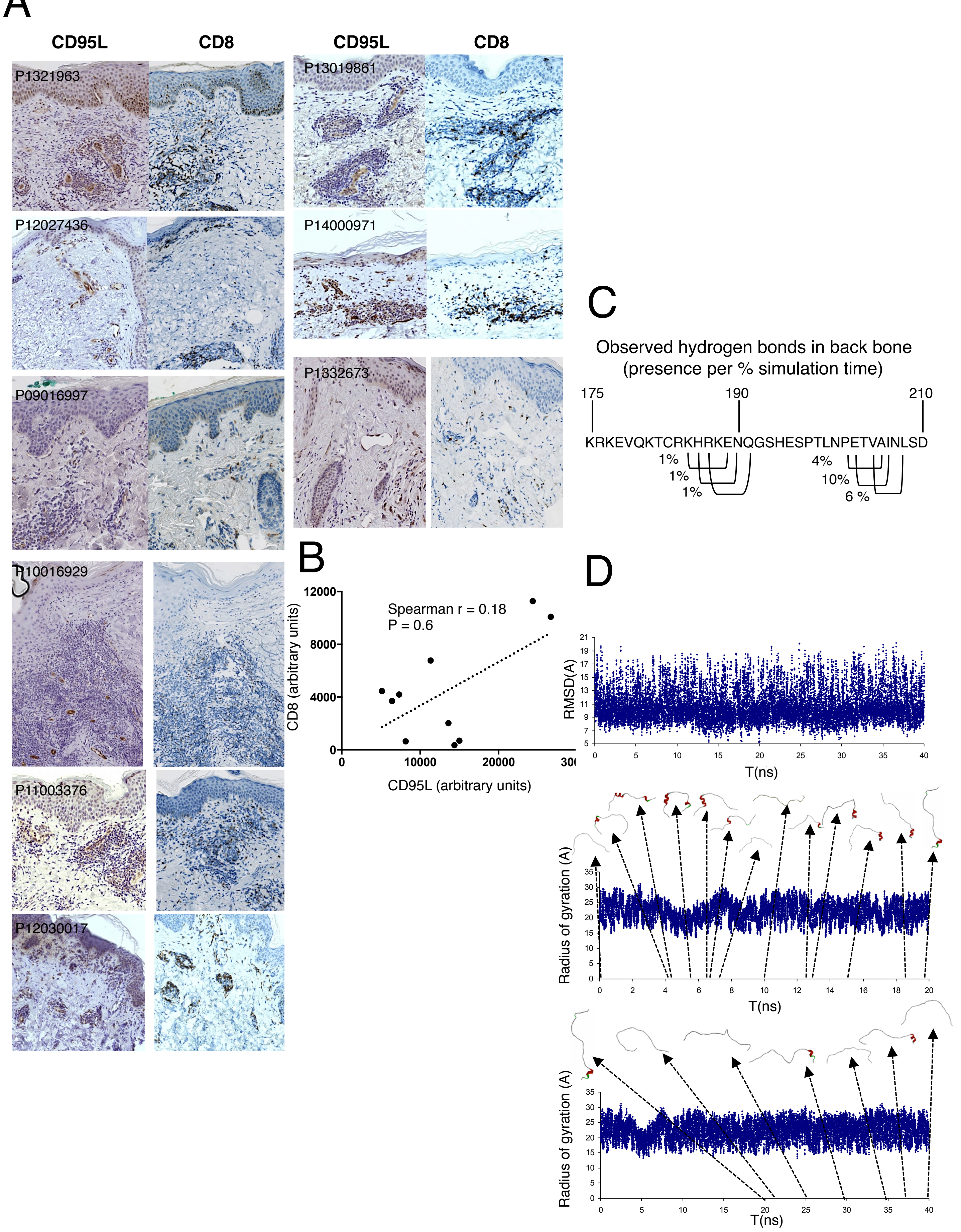

SUPPORTING TABLE 3

	interaction energy (kcal/mol)	van der Waals energy (kcal/mol)	Ligand Contact Surface Area (Å)	Receptor Contact Surface Area (Å)	RMSD vs 1YWO all heavy atoms (Å)	RMSD vs 1YWO backbone atoms (Å)
**Predicted SH3-PLCγ1 / CID-CD95 complex (TCRKHRK peptide)**						
**protein protein docking**	-122	-26.5	203	200	na	na
**homology modelling**	-143	-24.4	225	222	na	na
**SH3-PLCγ1 / SLP-76 complex (PPVPPQR peptide)**						
**PDB : 1YWO**	-118	-35	238	229	0	0
**redocking (pose 6)**	-90	-40	238	232	6.6	5.1
**redocking (pose 29)**	-118	-37	243	233	2.2	2.2
**protein protein docking (pose 10)**	-98	-36	220	218	5.5	3.6
**protein protein docking (pose 20)**	-138	-30	236	213	3.9	2.5
